# Strong, Shape-Memory Lignocellulosic Aerogel *via* Wood Cell Wall Nanoscale Reassembly

**DOI:** 10.1021/acsnano.2c11220

**Published:** 2023-01-30

**Authors:** Jonas Garemark, Jesús E. Perea-Buceta, Martin Felhofer, Bin Chen, Maria F. Cortes Ruiz, Ioanna Sapouna, Notburga Gierlinger, Ilkka Antero Kilpeläinen, Lars A. Berglund, Yuanyuan Li

**Affiliations:** † Wallenberg Wood Science Center, Department of Fiber and Polymer Technology, KTH Royal Institute of Technology, SE-10044Stockholm, Sweden; ‡ Materials Chemistry Division, Department of Chemistry, Faculty of Science, University of Helsinki, 00560Helsinki, Finland; § Department of Nanobiotechnology, Institute of Biophysics, University of Natural Resources and Life Sciences, 1190Vienna, Austria; ∥ Division of Glycoscience, Department of Chemistry, KTH Royal Institute of Technology, AlbaNova University Centre, 106 91Stockholm, Sweden

**Keywords:** aerogel, wood, cell wall reassembly, shape-memory, strong

## Abstract

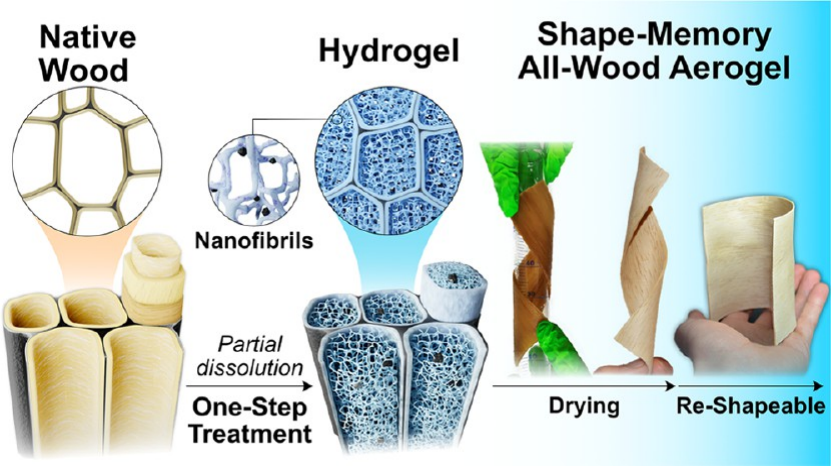

Polymer shape-memory aerogels (PSMAs) are prospects in various fields of application ranging from aerospace to biomedicine, as advanced thermal insulators, actuators, or sensors. However, the fabrication of PSMAs with good mechanical performance is challenging and is currently dominated by fossil-based polymers. In this work, strong, shape-memory bio-aerogels with high specific surface areas (up to 220 m^2^/g) and low radial thermal conductivity (0.042 W/mK) were prepared through a one-step treatment of native wood using an ionic liquid mixture of [MTBD]^+^[MMP]^−^/DMSO. The aerogel showed similar chemical composition similar to native wood. Nanoscale spatial rearrangement of wood biopolymers in the cell wall and lumen was achieved, resulting in flexible hydrogels, offering design freedom for subsequent aerogels with intricate geometries. Shape-memory function under stimuli of water was reported. The chemical composition and distribution, morphology, and mechanical performance of the aerogel were carefully studied using confocal Raman spectroscopy, AFM, SAXS/WAXS, NMR, digital image correlation, *etc.* With its simplicity, sustainability, and the broad range of applicability, the methodology developed for nanoscale reassembly of wood is an advancement for the design of biobased shape-memory aerogels.

## 1 Introduction

Polymer shape-memory aerogels (PSMAs) combine the feature of polymers to recover to their original state from a temporary state under stimuli and aerogel structural advantages of high porosity and specific surface area (SSA). These materials are receiving increasing attention as advanced thermal insulators for aerospace applications and as actuators and sensors for soft robotics and biomedicine. The sustainability issues associated with fossil polymers for PSMAs have driven the development of biopolymer-based SMAs. Cellulose is a promising candidate given the enormous annual production (about 7.5 × 10^10^ tons),^1,2^ carbon neutrality (storing CO_2_ during cellulose formation), high crystal modulus (137 GPa),^3^ and the potential for multifunctionalization.

One successful example is nanocellulose-based SMAs which have been intensively studied, including shape-memory behavior under the stimuli of water^4^ and electrical charge.^5^ Jiang *et al.* obtained shape-memory nanocellulose aerogels by cyclic freezing–thawing of TEMPO-oxidized nanocellulose gels.^6^ Strong associations between nanocellulose fibrils were formed through cyclic freezing–thawing; however, the structural integrity of the nanocellulose aerogel was a problem under aqueous conditions. Gu *et al.* followed the work and prepared shape-memory cellulose nanofibril aerogels by *in situ* deposition of Pd nanoparticles; nevertheless, the mechanical properties remained a problem.^7^ Chemical cross-linking or introduction of second-phase polymers offers effective solutions.^8^ Kim *et al.* obtained cross-linked nanocellulose SMAs by maleic acid functionalization of nanocellulose followed by sodium hypophosphite cross-linking and freezing-drying, which improved the mechanical stability in both wet and dry conditions.^4^ Li *et al.* introduced polyethylenimine (PEI) into nanocellulose aerogel to form stable networks through electrostatic interaction, resulting in wet stable SMAs with shape recovery properties under stimulation of water.^9^ Even with current successes, nanocellulose extraction is energy- and time-demanding, limiting further development. Additional physical or chemical modifications of the nanocellulose or the aerogel further complicate processing which might contradict green chemistry guidelines.

Obtaining cellulosic aerogels directly from wood where nanocellulose already is hierarchically organized and distributed in a hemicellulose and lignin matrix is an attractive alternative. In addition, wood is mechanically stable in aqueous solutions, which is associated with the lignin-containing structure.^10^ In earlier works, porous wood was obtained through delignification followed by hemicellulose removal.^11−15^ Wang *et al.* reported a shape-memory property of such porous wood, in which the wood shape recovered after compression by absorption of water, but only in the tangential direction.^16^ The absence of lignin and hemicelluloses in the porous wood showed substantial material losses during fabrication (reaching up to 65 wt %),^11^ limited mesopores, low specific surface area (SSA, <50 m^2^/g), and inferior structural integrity. Strategies of introducing an extra polymer network such as polyacrylamide^17,18^ or *N*-isopropylacrylamide^19^ in the lumina space were further developed to improve mesoporosity and structural integrity. Nevertheless, polymer diffusion into wood is a considerable challenge. Recent progress showed wood aerogels fabrication through careful nanoscale reassembly of the cell wall components in the space of empty lumina (20–30 μm).^20^ Initial success was demonstrated by partial cell wall dissolution and regeneration through DMAc/LiCl^21^ or ionic liquid (IL)^22^ treatment of delignified wood, resulting in anisotropic wood aerogels with nanofibrillated networks filling the lumina. Further maintaining lignin as an interfiber bonding agent in the structure enhanced mechanical strength significantly while simplifying the process by eliminating the delignification step.^23^ However, the shaping of such wood aerogel is limited and the shape-memory function attempted here has never been reported.

**Figure 1:**
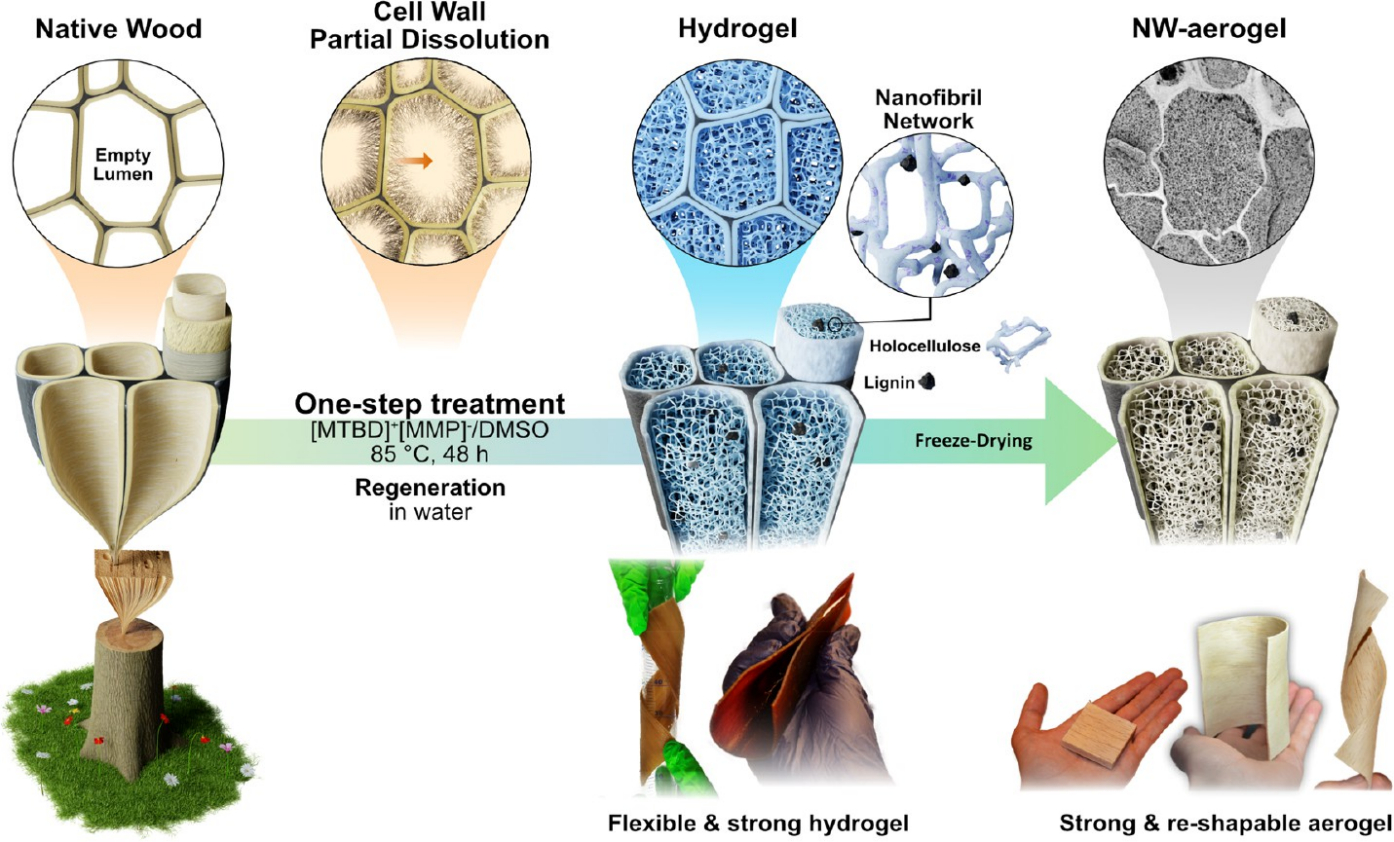
From the left, schematic illustration showing the hierarchy of natural wood and the one-step treatment to form highly malleable wood hydrogels. The hydrogels show holocellulose nanofibrillated networks in the cell lumina with lignin particles decorating the networks. On the right-hand side, through freeze-drying the hydrogel an aesthetic aerogel similar to natural wood is achieved but has a substantially different nanostructure.

In this work, strong and shape-memory all-wood aerogels were prepared in a one-step chemical treatment of native wood using the IL electrolyte mixture of [MTBD]^+^[MMP]^−^/DMSO as the solvent system (Figure 1). The wood cell wall composition was maintained, and the components were redistributed to the empty lumina as lignin-containing nanofibril networks. Highly flexible hydrogels were formed, giving an opportunity to reshape the aerogel to various geometries. Shape-memory properties were observed so that the aerogel could return to its original shape repeatedly, upon submerging into water. Confinement of nanofibril networks within the lumina and preserved lignin in the remaining cell walls led to a special combination of high strength (5.1 MPa), specific surface area (up to 220 m^2^/g), and low radial thermal conductivity (0.042 W/mK). This synthesis route contributes significantly toward more sustainable development of advanced hydrogels and aerogels in high yield from renewable resources.

## 2 Results and Discussion

### 2.1 Synthesis and Morphology

**Figure 2:**
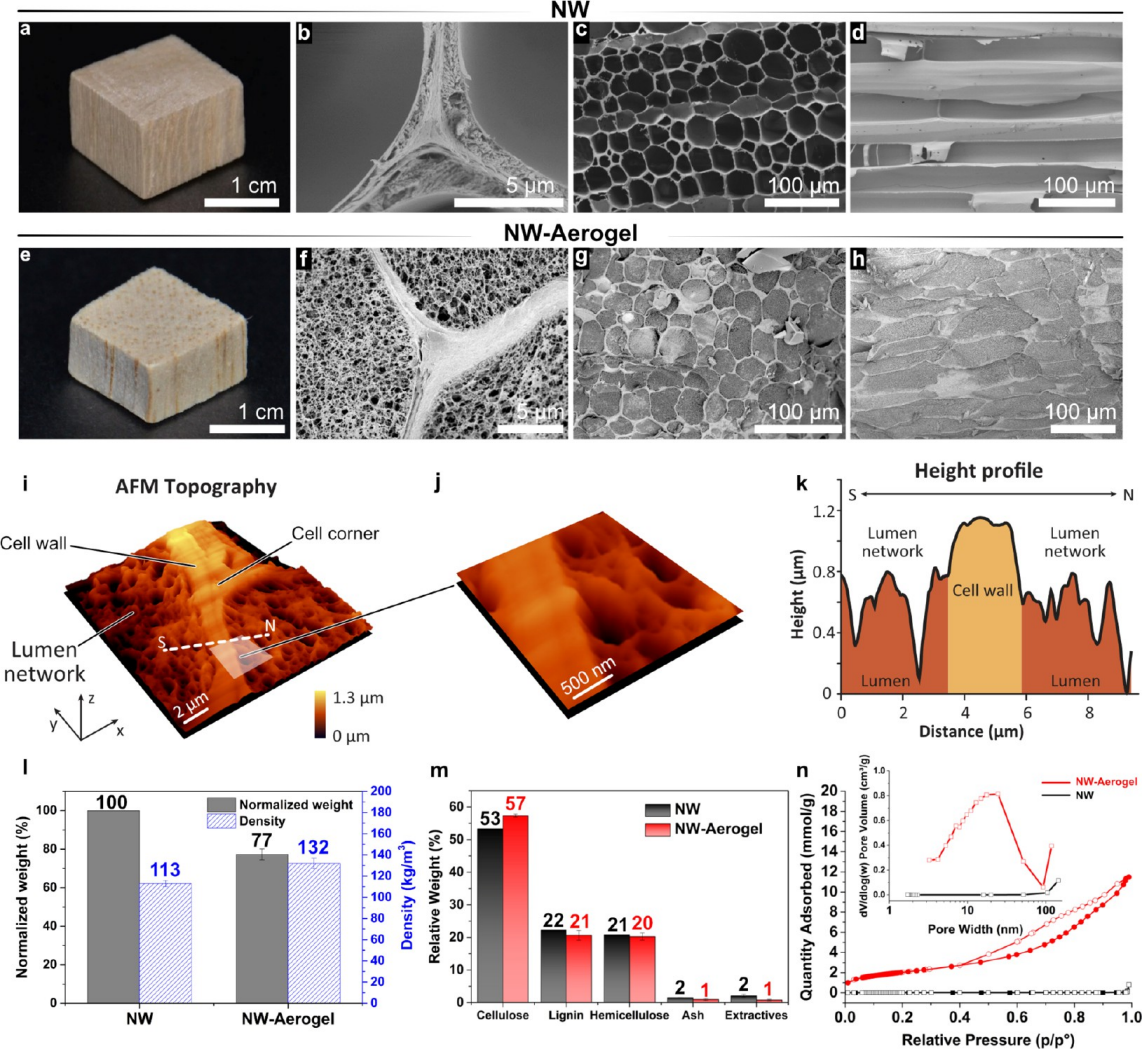
(a) Photograph of NW, (b) high magnification SEM image, (c) low magnification SEM image of cross-section, and (d) low magnification SEM image of the surface along the fiber direction of NW. (e) Photograph of NW-Aerogel, (f) high magnification SEM image, (g) low magnification SEM image of cross-section, and (h) low magnification SEM image of the surface along the fiber direction of NW-Aerogel. (i) AFM topography image of NW-Aerogel with remaining cell wall and cell corner. (j) Zoom in to the interface between the cell wall and network. (k) Height profile along the dotted line in panel a, showing the different sizes of the nanostructures and the cell wall interphase. (l) Weights and densities of NW and NW-Aerogel. (m) Carbohydrate, lignin, ash, and extractive contents of NW and NW-Aerogel followed by (n) nitrogen physisorption isotherms and inset image of the pore-size distributions of NW and NW-Aerogel. The SEM and AFM images are based on freeze-dried samples.

Shapeable all-wood aerogels were prepared *via* a one-step chemical treatment by submerging native wood (NW, Figure 2a–d) in an organic electrolyte solution containing 20 wt % of the ionic liquid [MTBD]^+^[MMP]^−^ and 80 wt % of the organic solvent DMSO for 48 h at 85 °C. With the help of the IL treatment, wood cell wall biopolymers were redistributed at the nanoscale resulting in a change of the structure (Figure 2e–h), while maintaining the original composition (Figure 2m).

Native wood is brownish with large empty lumina (Figure 2a–d). After the one-step IL treatment, the wood color remains (Figure 2e), yet with the nanostructure greatly transformed. Nanofibril networks are formed and homogeneously fill the lumen space (Figure 2f–h). The porous structure of the lumina and the cell wall lumina network interphase was further verified by atomic force microscopy (AFM). As shown in Figure 2i–k, the network attaches distinctly to the remaining cell wall, which strongly supports our assumption of the dissolution and conformational change of the cell wall. This cell wall dissolution is apparent from the interface between the fibril network and the cell wall (Figure 2f,j and Supporting Information Video S1). Interestingly, the lignin-rich cell corner and middle lamella are still clearly distinguishable (Figure 2f,i). During this processing, 23 wt % weight loss was observed mainly due to the diffusion of dissolved cell wall materials from the wood structure to the IL solution (Figure 2l). Nevertheless, a slight density increase from 113 (NW) to 132 kg/m^3^ (NW-Aerogel) was noticed due to shrinkage upon regeneration. Intriguingly, the carbohydrate, lignin, ash, and extractive analysis showed relatively unchanged chemical composition (Figure 2m). Lignin, hemicellulose, and cellulose contents of the NW-Aerogel were 21, 20, and 57 wt %, respectively, which are similar to those of NW (22, 21, and 53 wt %, respectively). A mass balance of the wood treatment can be seen in Table S1 of the Supporting Information.

The redistribution of biopolymers and the formation of nanofibrillar networks in lumen space resulted in increased specific surface area (SSA). In Figure 2n, nitrogen physisorption of critical point dried (CPD) NW-Aerogel showed a type-IV isotherm. The large hysteresis loop of the desorption curve indicated capillary condensation in open-ended mesopores. The significant slope between 0.05 < *P*/*P*_0_ < 0.4 suggested a high SSA with a value up to 220 m^2^/g, which is about 200 times larger than NW (1 m^2^/g). According to the pore size distribution (Figure 2n), mesopores dominate in the aerogel as a large signal was seen at 10–30 nm. Freeze-drying (FD) was also performed, showing pore dominance at around 30 nm; see Figure S1. This is consistent with the nanoporous structure observed by SEM images (Figure 2f–k). The SSA for FD aerogel is ∼20 m^2^/g, much lower than CPD aerogel. The reason for such a large discrepancy of SSA is that drying from low surface tension fluid (supercritical CO_2_) retains the nanoporosity of the wet specimen well, whereas the freezing process in FD can lead to aggregation of substance due to ice crystals growth and some loss in nanoporosity, depending on freezing rate.^24^

### 2.2 Shape-Memory and On-Demand Shaping Properties

**Figure 3:**
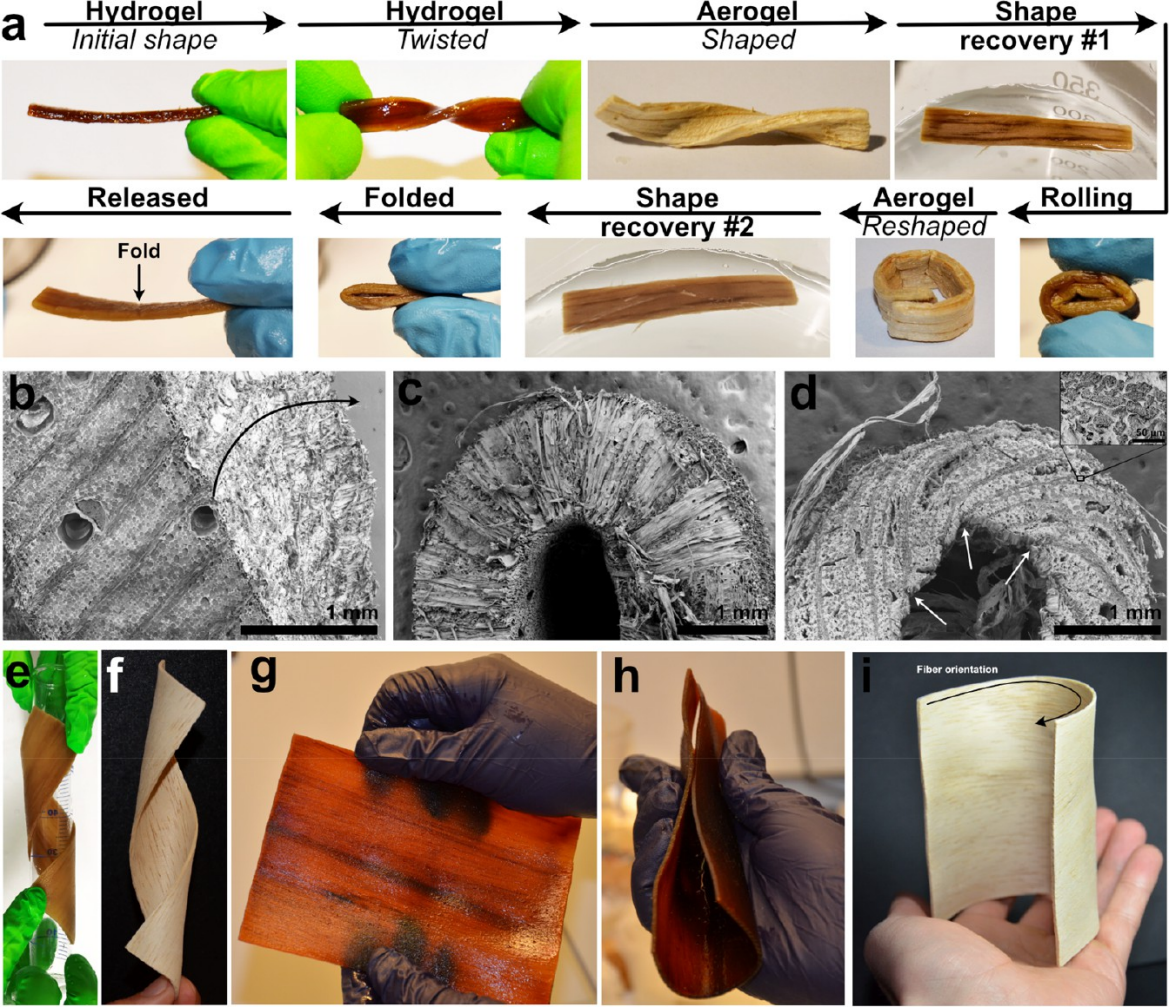
(a) Photographs of wood hydrogel, twisted hydrogel, its dry shape, shape-memory behavior, and reshaped structure. NW-Aerogels folded against (b) longitudinal, (c) cross-sectional, and (d) radial directions with an inset SEM image of the fibrillated lumen networks. (e) Wood hydrogel wrapped around a glass cylinder and (f) subsequent aerogel of helical shape. (g) Large wood hydrogel veneer, (h) deformed state, and (i) its subsequent dry hydrogel, with u-bend against fiber direction.

The reassembly of wood biopolymers led to a significant change in physical properties. Excellent deformability was observed for the hydrogel prior to drying. It was twisted, bent (along the fiber direction or perpendicular to the fiber direction), and rolled, as shown in Figure 3a. This must be related to redistribution of the stiff cellulose polymers of the secondary cell walls and a structure change to lignin which occupies the cell corners (CCs) and compound middle lamella (CML) between fibers. It should be noted that this deformability could be exploited to attain aerogels of various geometries (Figures 1 and 3a,e–i). In addition, the NW-Aerogel, after drying, showed shape-memory properties as it returned to the initial shape when it was soaked in water. Figure 3a shows that a twisted NW-Aerogel could return to the initial shape and be further rolled and folded. The stimulus to water can be seen in Video S2. To the best of our knowledge, this shape-memory property of wood aerogel has never been reported. Panels b–d of Figure 3 are SEM images of NW-Aerogel, bent NW-Aerogel, and rolled NW-Aerogel, showing the deformation of aerogels and the preserved nanofibril network during the processing. No apparent cracks were noticed during the multiple shapings along or against the fiber direction, although subfolds can be seen from buckling (arrows in Figure 3d). A similar shape-memory phenomenon could be achieved even with ambient drying followed by exposure to water; see Figure S2. The shape memory property makes the aerogel attractive as a sensor.^25,26^ The processing and properties apply to large samples. Figure 3e shows a larger hydrogel (length, 160 mm; width, 40 mm; thickness, 2 mm) which could be wrapped around a glass cylinder and subsequently dried to obtain a helix-shaped aerogel. Panels g and h of Figure 3 show an even larger hydrogel sample (length, 160 mm; width, 100 mm; thickness, 2 mm) with high deformability in all directions. Subsequently, a large NW-Aerogel with u-shape could be obtained (Figure 3i). The mechanism for the memory effect must be related to recoverable deformation of the cellulose nanofibril structures. Although global deformation is very large, the strain at the scale of a nanofibril can be small for a porous material.

### 2.3 Chemical and Structural Analysis

**Figure 4:**
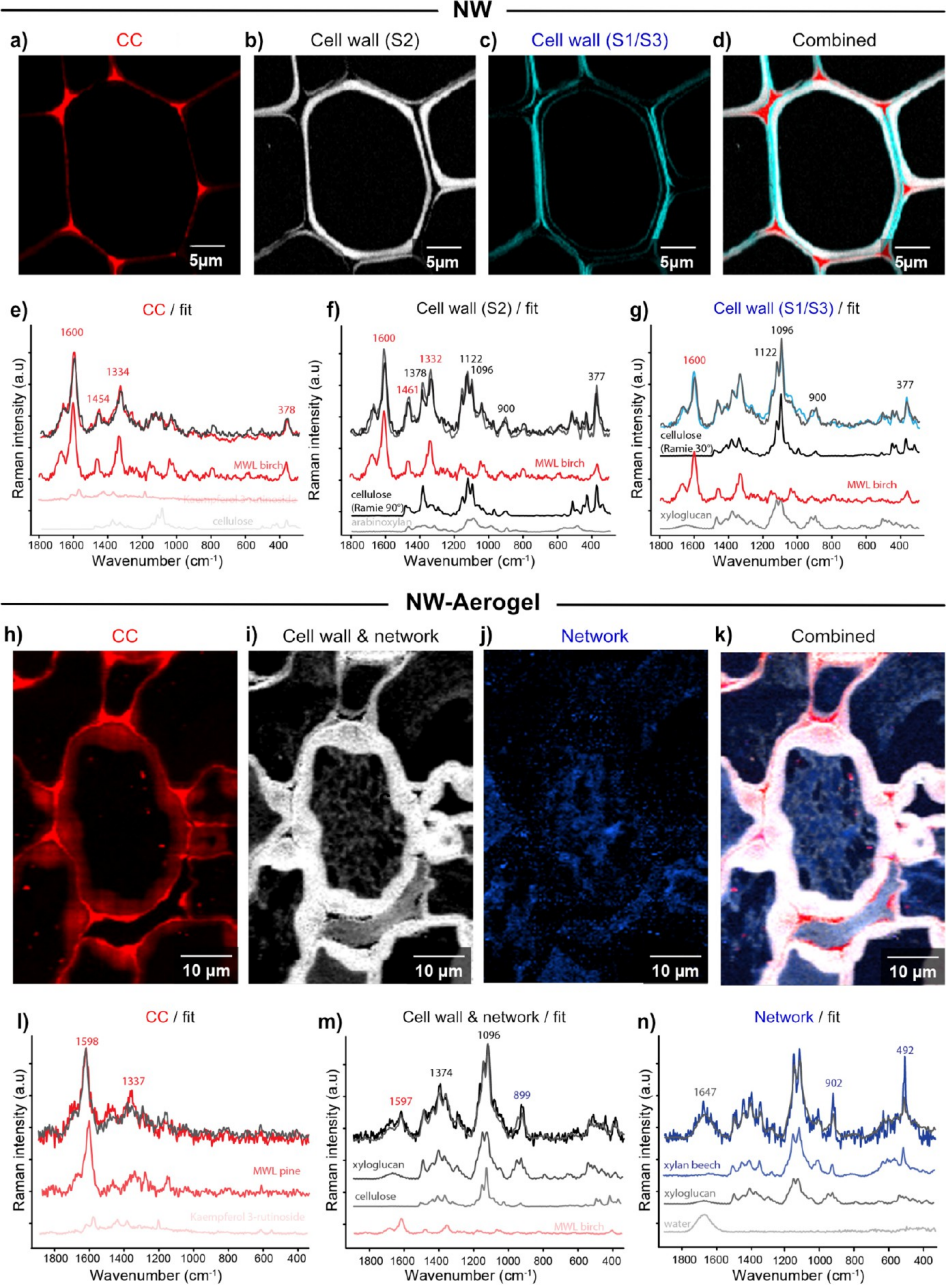
Confocal Raman spectroscopy images of (a–d) NW and (h–k) NW-Aerogel, followed by the fitted spectra of (e–g) NW and (l–n) NW-Aerogels. For NW, the most different spectra were derived from cell corner (CC, a) with almost pure lignin composition (e, CC spectrum fitted by milled wood lignin birch), cell wall (S2, b) composed of lignin (f, red spectrum), cellulose aligned along the fiber (f, blue; Ramie fiber axis laser polarization, 90°) and hemicellulose (f, gray), and the cell wall S1/S3 (c) distinguished due to higher cellulose microfibril angle (g; cellulose Ramie, 30°). For NW-Aerogel, lignified CC and a lignin gradient in the cell wall visualized (h) and a change in lignin composition (l, fit with milled wood lignin pine). Cell wall and the network (i) and their common carbohydrate-rich spectral signature (m). Lumen network (j) rich in hemicellulose with typical bands at 492 and 902 cm^–1^ (n).

The high SSA and shape-memory properties are related to the aerogel nanostructure. To understand the structure changes, Raman spectroscopy was used to probe the chemistry of the NW-Aerogel on the micro- and nanoscales (Figure 4).

Sections of native and treated balsa wood were scanned to compare the chemical composition of the NW (Figure 4a–g) and the NW-Aerogel in context with the microstructure (Figure 4h–n). Using multivariate approaches, we first searched for the most distinct Raman spectra to visualize changes in chemistry (Figure 4a–d,h-k). In a second step, the interpretation of the spectra was verified based on a linear combination of reference spectra (Figure 4e–g,l–n). In NW, the CC (Figure 4a) was separated from the secondary cell wall S2 (Figure 4b) and the S1 and S3 layer (Figure 4c). Fitting the representative experimental spectra with reference spectra (carbohydrates and aromatics), the results verify the lignin nature of the CC in NW (Figure 4a) by the reference “*milled wood lignin (MWL) birch*” (red spectrum in Figure 4e). The cell wall spectra showed the multicomponent nature of the cell wall at any pixel (Figure 4f,g, lignin, cellulose, and hemicellulose). The two cell wall spectra (S2 and S1/S3) were differentiated due to changes in the cellulose microfibril angle, as verified by a fit with Ramie 90° in S2 *vs* Ramie 30° in S1/S3 (Figure 4f,g). This means cellulose fibrils are oriented parallel to the fiber axis in the S2 layer (90° in the cross-section) and a microfibril angle around 30° (60° in the cross-section) dominates in S1 and S3.^27^ Additionally, hemicelluloses contribute to the Raman signal of the cell walls S2 and S1/S3 (Figure 4f,g, gray spectra).

In the NW-Aerogel, the multivariate algorithm separated the CC (Figure 4h) from the cell wall (Figure 4i) and the nanofibrillated networks in the lumen (Figure 4j). Fitting with references verified the lignin composition of the CC (Figure 4l) and the spectral fitting with “*MWL pine*” points to a lignin structure with less methoxy groups (Figure 4h). Lignin content gradually decreases toward the lumen, and some particles were found in the lumen networks (Figure 4h). The cell wall and lumen network have the same spectral signature (Figure 4i) and are carbohydrate-rich with a lower amount of lignin (Figure 4m). The fibrillated lumen networks were distinguished (Figure 4j) with typical bands at 492 and 902 cm^–1^ and fit with the hemicellulose xylan (Figure 4n). Altogether, nanofibrillated networks composed of holocellulose (cellulose and hemicellulose) were visualized, while cell walls showed a gradual lignin increase from the surface toward the cell wall interior.

The combined chemical image shows again the lignin gradient in the secondary cell wall (Figure 4k), and the holocellulose nanofibrillated networks in the lumen contain dispersed lignin particles. The secondary cell walls were either thick and swollen or nearly fully dissolved; intact CC and CML were seen in all cases. A larger area Raman image is presented in Figure S3 and a focus on the cell wall in Figure S4. The still lignified CC results in good interfiber adhesion, and the “aerogel” filled lumen provides enhanced specific surface area. This dual structure has a positive effect on both strength and fibril surface accessibility, a rather rare combination for porous materials.

**Figure 5:**
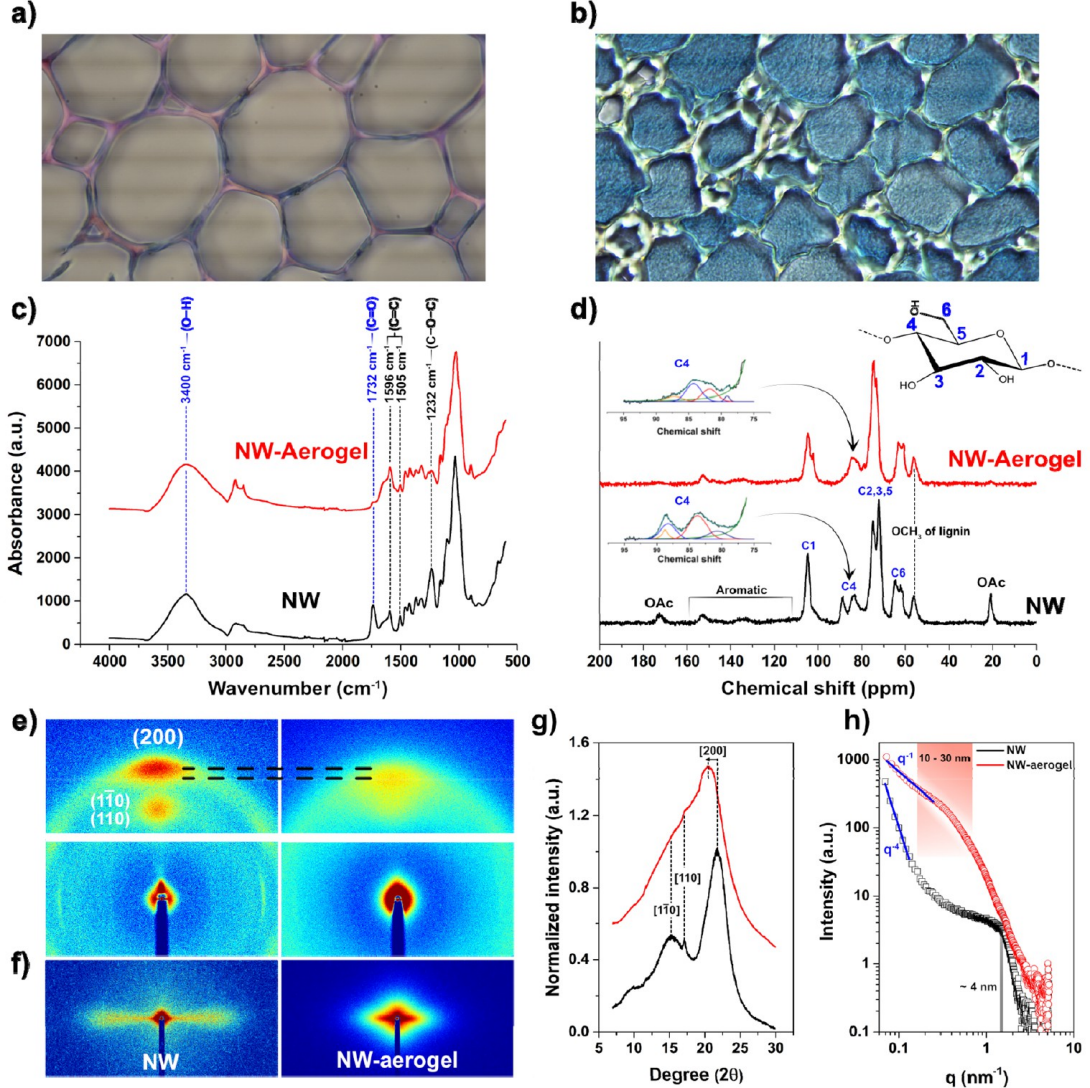
(a) FCA-stained NW and (b) stained NW-Aerogel. (c) FT-IR data of NW and NW-Aerogel, (d) ^13^C solid-state NMR, (e) wide-angle X-ray scattering (WAXS), (f) small-angle X-ray scattering (SAXS) images, (g) WAXS diffractogram, and (h) SAXS diffractogram of wet NW and never-dried NW-Aerogel.

The high carbohydrate content of lumen networks in NW-Aerogel was further confirmed by histochemical staining using Fuchsin–Chrysoidin–Astra (FCA) (Figure 5a,b). The basic Fuchsin stains lignin red and the astra blue acts by staining carbohydrates blue (Figure 5a).^28^ In the NW, colors are concentrated in the cell wall, since lumen is empty. In the NW-Aerogel, the lumen networks and the outer parts of the cell walls exhibit an intense homogeneous blue color (Figure 5b), characteristic of a high concentration of carbohydrates. The structural homogeneity was exceptional even in large sections (Figure S5). There was little to no red coloration in the inner intercellular corners, which might be due to lignin modification. NMR analysis of the [MTBD]^+^[MMP]^−^/DMSO treated milled wood lignin (MWL) showed a more degraded lignin structure as the abundant β-O-4′ linkage decreased from 65% of NW to 54% (Figure S6).

Cellulose and lignin are inherently rigid structures. Aerogel flexibility is related to the fine nanofiber diameter, but changes to the physical and chemical biopolymer structures may also contribute. Fourier transform infrared spectroscopy (FT-IR) was applied to identify changes in chemical bonds. Comparing NW and NW-Aerogel, lignin retention was evident as aromatic C=C stretching vibrations (1596 and 1505 cm^–1^)^29^ are present in the NW-Aerogel spectra. The ratio between 1596 and 1505 cm^–1^ changed, and the signal associated with C–O–C stretching in phenol ether bonds (1232 cm^–1^) decreased.^30^ The reduction of 1232 and 1505 cm^–1^ signals was reported as a cause of breaking the β-O-4′ linkages, which aligns with the observed decrease of β-O-4′ linkages detected from NMR of the NW-Aerogel’s lignin (Figure S6). The intensity for the carbonyl stretching vibration (1732 cm^–1^) was considerably decreased, mainly due to loss of hemicellulose acetyls after IL treatment.^31^ This was further supported by decreased wood surface charge from 139 μeq/g of NW to 111 μeq/g for NW-Aerogel since carboxylic acid in hemicelluloses mainly contribute to the surface charge (Table 1). The cellulose chemical structure did not change much, as expected. Peaks corresponding to asymmetric bridge stretching vibration of the β-glycosidic bonds (893 cm^–1^),^32,33^ skeletal vibrations of glucopyranoside rings (1034 cm^–1^),^32,33^ and asymmetric stretching of C–O–C (1150 cm^–1^)^34^ remained similar. A slight increase in intensity was observed for the stretch vibrations of hydroxyl groups (3400 cm^–1^) and stretch vibrations of C–H (2900 cm^–1^).

**Table 1:** Summary of Physical Properties of NW and NW-Aerogel

	NW	NW-Aerogel
Density	112.8 ± 3.4	132.4 ± 5.4
Porosity (%)	92.5 ± 0.2	91.2 ± 0.4
SSA, FD (m^2^/g)	–	19.4 ± 1.8
SSA, CPD (m^2^/g)	–	193.9 ± 26.6
Av pore diam (nm)	104.3 ± 47.2	26.3 ± 4.3
Total charge (μeq/g)	139.2 ± 5.5	110.1 ± 11.7
Mechanical Properties (Wet State)		
Tensile strength, wet, σ_L_ (MPa)	7.92 ± 1.96	1.74 ± 0.64
Tensile modulus, wet, σ_L_ (MPa)	353 ± 81.5	10.16 ± 2.16
Mechanical Properties (Dry State)		
Yield strength, dry, σ_L_ (MPa)	6.63 ± 0.98	5.13 ± 1.05
Yield strength, dry, σ_R_ (MPa)	0.51 ± 0.01	0.61 ± 0.07
DIC: Young’s modulus, dry, *E*_L_ (MPa)	1213 ± 155	1057 ± 25
Young’s modulus, dry, *E*_L_ (MPa)	153 ± 29	154 ± 27
Young’s modulus, dry, *E*_R_ (MPa)	161.7 ± 29.3	60.4 ± 14.68

**Figure 6:**
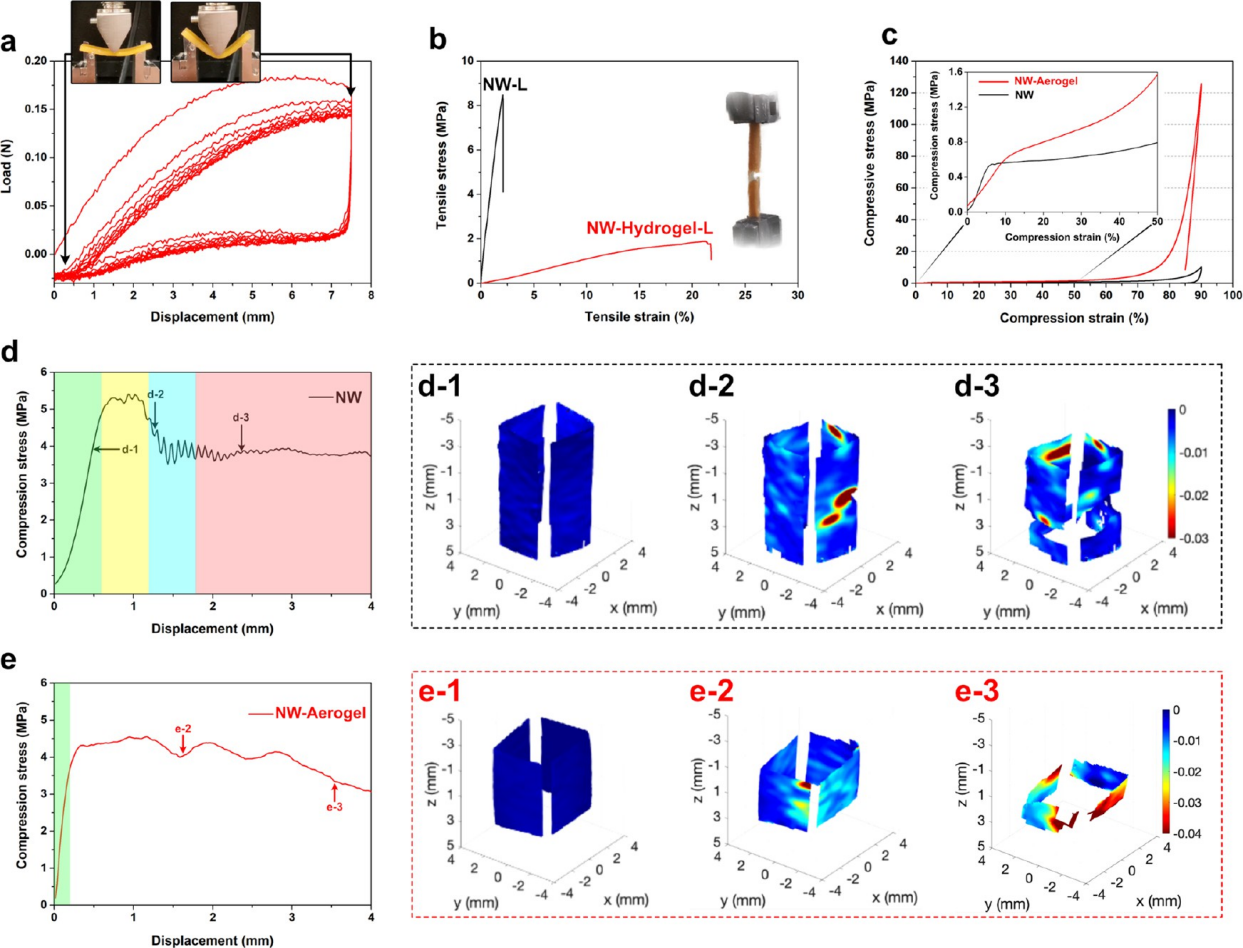
(a) Three-point bending test on NW-Aerogel. (b) Tensile test of wet NW and NW-hydrogel in the longitudinal direction of wood. (c) Typical curves for radial compression of NW-Aerogel and NW (inset, magnification of the lower range strains). (d and e) Digital image correlations of samples compressed in the longitudinal wood direction, in which strain fields at different displacements for (d-1 to d-3) NW and (e-1 to e-3) NW-Aerogel are shown.

Dissolution and regeneration of cellulose alter the native cellulose I structure in NW and influence mechanical properties. Crystalline changes were studied with ^13^C solid-state NMR. Through deconvolution of the C4 signal, the cellulose crystallinity was discernible. The crystalline C4 signal at 88 ppm diminished and the crystallinity index decreased from 43% of NW to 23% of NW-Aerogel (Figure 6d). Hence, disordered cellulose dominates the cellulose structure in the NW-Aerogel. An upfield shift of the C-6 resonance in the NW-Aerogel (64.9 to 63.2) was observed which may correspond to the decrease in crystallinity,^35,36^ although this shift has also been reported in association with cellulose II.^37,38^ Intense signals for methoxyls and aromatics from lignin were observed at 56 ppm and between 110 and 155 ppm for both NW-Aerogel and NW,^39^ which agrees with the FT-IR and carbohydrate analysis data. The signal for hemicellulose acetyl groups (20.7 ppm) vanished in NW-Aerogel, which aligns with FT-IR data, further justifying deacetylation.^39^ A small peak is also seen around 78–79 ppm, which could be a hemicellulose (xylan) signal.^40^ The significant reduction in crystallinity coupled with the microscopic redistribution of biopolymers may contribute to the reduced stiffness and increased flexibility of the hydrogel after treatment. The small-scale changes in the CC and CML will influence the nature of interfiber bonding. Physical and chemical changes in this region are surely important and contribute to increased flexibility, as well as the increased porosity of the cell wall. Apparently, the nature of interfiber bonding in treated hydrogels is not degraded by large global deformation of the materials.

Partial wood dissolution and redistribution of constituents lead to a change in orientation of the inherent cellulose polymers, indicated by the wide-angle X-ray scattering (WAXS) data. The degree of orientation was resolved through azimuthal integration of the (200) reflections of the WAXS images in Figure 5e. NW and NW-Aerogel showed high orientation degrees of 0.88 and 0.81, respectively. The nanofibril lumen network fraction of the NW-Aerogel is near random in space (Figure 2f and 4i), so that the average cellulose orientation is reduced. The decrease, however, is relatively moderate given the high fibrillation density. Possibly, a significant amount of the network originates from cellulose of nonvertical orientation (S1/S3 layer) and associated hemicellulose (Figure 4j and 4n). The S2 layer is the main contributor to cellulose orientation, where nanofibril alignment was preserved (Figure 4i). In the NW-Aerogel, the orientation is mainly from disordered cellulose as the cellulose (200) reflection shifted to lower *q*-values (Figure 5e,g).

Long-range structural information at the nanoscale (1–100 nm) was studied using small-angle X-ray scattering (SAXS). The 2-D scattering of Figure 5f shows an anisotropic scattering of NW, while for NW-Aerogel, mixed random and anisotropic scattering was seen. This is due to the generation of nanopores in the remaining cell walls and nanofibrils formed in the lumina of NW-Aerogel. Figure 5h shows the SAXS data of intensity *vs* scattering length (*q*). NW has a distinct signal at 1.58 nm^–1^ representing the correlation distance of cellulose nanofibrils (around 4 nm) within the wood cell walls.^41−43^ A small remaining signal indicative of the microfibril correlation length was observed for NW-Aerogel.^42,44^ From the Kratky plot in Figure S7 comparing NW and NW-Aerogel, the signal is more prominent. A broad peak is present around 0.2–0.4 nm^–1^ for NW-Aerogel, indicating pores or fibrils of 10–30 nm, in agreement with the BJH pore-size distribution (Figure 2k). The dominating nanostructure signal of NW-Aerogel is clear from the Kratky plot in Figure S7. In the low *q* region, a slope of *q*^–4^ signifies surfaces of large pores and fiber lumina for NW. A significant change is seen for NW-Aerogel as the slope decreases to *q*^–1^ representing a rod-like structure assigned to the nanofibrillated networks of fiber lumina.

Based on the chemical structure analysis, the partial dissolution/regeneration and structure change were understood as follows. The ionic liquid solution [MTBD]^+^[MMP]^−^/DMSO has an amphiphilic character of high basicity (hydrogen bond accepting ability),^45^ enough to dissolve cellulose by the interaction of the polar functionalities and hydrophobic domains. The primary dissolution driving force is the entropic gain of polymer in solution.^46^ However, crystalline cellulose fibrils exist in the cell wall surrounded by a compact matrix of hemicellulose and lignin. Hence, it is understood that swelling and partial dissolution of lignin are necessary to facilitate diffusion and interaction with cellulosic compounds. Precipitated lignin (Figure 4h) within the cellulosic networks appoints to high lignin dissolution, and the mass balances (Table S1) for NW and NW-Aerogel indicate a nonselective dissolution of the wood cell wall. Solubilized biopolymers diffuse from the compact cell wall to the lumen bulk solution and precipitate when submitted to an antisolvent (water). Precipitation occurs as solvent molecules are replaced by antisolvent molecules, wherein re-formation of the biopolymer supramolecular structures transpires.^47^ The redistribution of the cell walls *via* partial dissolution and regeneration leads to nanoporous networks constituting amorphous cellulose (Figure 5d,g) and hemicellulose within the cell lumina (Figure 4j), highly porous secondary cell walls (Figure 4i), and a modified lignin structure of the CML and CC. The regenerated lignin structure is likely more porous and showed fewer β-O-4′ linkages (from 65% to 54%; see Figure S6) ,which reduces its stiffness, yet retains the interfiber bonding function. Cellulose lost the correlation distance (∼4 nm) between fibrils and instead shows enlarged nanopores (∼30 nm), which likely are more prone to wetting; see Figure 5h. The regenerated cellulosic lumen networks and secondary cell walls of NW-Aerogel are highly hydrophilic with a significantly higher surface area for interaction compared to NW.

### 2.4 Mechanical Properties

To understand the deformability and mechanical performance of the wood hydrogel and aerogel, careful mechanical property characterization was performed. Three-point bending tests were performed in several cycles to assess the flexibility and changes in behavior. In general, the NW-Hydrogel is highly deformable and flexible (Figure 6a). The NW-Hydrogel shows nonlinear behavior with strong hysteresis effect (unloading behavior provides lower deformation work than loading). After the stiffer behavior during the first cycle, some structural changes result in lower stiffness in the second cycle, although stiffness losses are then limited in the following cycles. In contrast, NW is stiffer and fractures at a displacement of ∼1.5 mm during the first cycle (Figure S8). Figure 6b shows the tensile stress–strain curves for NW and NW-Hydrogel samples in the longitudinal direction. NW-Hydrogel is more extensible with strain to failure (21%) 1 order of magnitude larger than for NW (2%), but at the cost of reduced tensile modulus (10.1 MPa) and ultimate tensile strength (1.74 MPa). The ductility is comparable to delignified wood infused with the ductile polymers such as polyacrylamide (PAAM)^17,18^ or *N*-isopropylacrylamide.^19^

Upon drying the hydrogel, the shape becomes fixed and shows similarities to NW. Figure 6c compares the stress–strain curves of NW and NW-Aerogel under radial compressive loading. Both samples were linearly compressed initially. The compressive modulus of NW-Aerogel (60.4 MPa) is obviously smaller than that of NW (161.7 MPa). In radial compression, cell wall bending is the main deformation mechanism.^48^ The main reason for lowered modulus is probably the conversion of cell walls into a highly porous state, lowering cell wall stiffness in bending. At higher strain, NW shows a plateau of constant stress typical for non-uniform plastic collapse of wood in radial compression,^48^ while NW-Aerogel shows significant strain hardening rather than a plateau. In NW-Aerogel, the stress increases monotonically, and this must be related to densification and stiffening of the nanofibril network in the lumen region. At high strain (>60%) cell walls and/or nanofibril networks form densified structures (cell walls/nanofibrils in close contact with neighbors) and stress increases rapidly. The stress of NW-Aerogel during densification greatly exceeds that of the NW despite similar porosities. The reason is the more homogeneous structure in the NW-Aerogel, which changes the scale of deformation mechanisms from >1 μm (cell wall bending and buckling) to well below micrometer scale (nanofibril network deformation).

From longitudinal compression tests (σ_L_) (Table 1), the yield strength was found to be 5.1 MPa for NW-Aerogel and 6.6 MPa for NW. This is more than 4 times the yield strength compared to our previously reported delignified wood aerogel.^22^ Li *et al.*(23) reported a wood aerogel based on balsa wood with 18 MPa longitudinal yield strength in compression, which is a consequence of significantly higher density compared to this work.^23^

Panels d and e of Figure 6 present the compressive stress–strain curves of NW and NW-Aerogel along the longitudinal direction. Panels d-1 to d-3 and e-1 to e-3 of Figure 6 show the compressive strain fields measured by mirror-assisted MV-DIC during loading.^49^ In the linear-elastic region (marked green in Figure 6d,e), the cell walls are compressed uniaxially for both samples, corresponding to the homogeneous strain field in Figure 6d-1,e-1. Thereafter, both samples show mainly constant global stress during deformation with some local strain concentrations, leading to progressive damage of the samples; see the strain fields in Figure 6d-2,e-2, and Video S3 and Video S4, respectively. From the end of linear deformation to the peak stress (yellow region in Figure 6d), the NW fibers develop small and almost invisible local concertina folds (plastic yielding) or kinking (Video S3). After the peak of the stress, in the blue region, the local small kinking coalesces and forms a single dominant kink band captured as local strain concentration (Figure 6d-2). The deformation of the fibers in the kink band is then stabilized due to the fiber lock-up from the volumetric constraints, leading to constant, stabilized stress. In the plateau region (red region in Figure 6d), the straight fibers at the edge of the kink band are crumpled and rotated, which broadens the kink band region; see Figure 6d-3. In the region without data, the strain is too large to be measured. It should be noted that the plateau region is significantly serrated, attributed to brittle cell collapse by end-cap fracture.^48,50^

In NW-Aerogel, no significant peak stress drop exists in contrast to NW. The nanofibril network in NW-Aerogel leads to more homogeneous microscale stress distribution and changes mechanisms of failure. Compared with the NW sample, the plateau stress of the NW-Aerogel does not change significantly. In the steady-state kink band region of the NW-Aerogel, the stress–strain curve is much smoother; see Figure 6e. The homogeneity of the strain field at larger scale is apparent in Figure 6e-2,e-3. Only small strain concentration exists in the center, while the kink bands exist mainly in the contact regions.

Based on the chemical structure and mechanical deformation understanding, the proposed mechanisms for shape-memory of the PSMA could be understood as follows. A general description of the shape-memory property can be made from the perspective of deformation energy, wherein the shape-memory effect relies on the storage and release of strain energy.^51,52^ In the original (dry) nondeformed state, the NW-Aerogel is in a low entropy and low strain energy state. When wetted, the water molecules readily diffuse and interact with the more accessible amorphous polymers, decreasing the polymer–polymer interactions and allowing for higher mobility. This leads to an increase in the entropy of the system. Water shows a plasticizing effect on NW-Aerogel, substantial swelling of the cell wall is observed from wet-state analysis (Figures 4k and 5b), and the material becomes highly flexible. Importantly, the wood hydrogel recovers elastically even after large deformation in the wet state (Figure 6a). Deforming the material in this wet condition and subsequently drying it result in a strained state of stored elastic energy.^53^ Upon drying, the plasticizing effect from water is lost and the polymers are less mobile, cellulosic polymers interact *via* secondary forces,^54^ and the material is locked in an elastically deformed state (high strain state) distinct to stiff and flexible nanofibril assemblies. Through rewetting in water, the biopolymers recover mobility,^55,56^ interfibril bonding is removed, system entropy increases, strain can be released, and the structure returns to its original shape.^51,52^ For polymer systems without nanofibrils, shape recovery is related to rubber elasticity effects and the glass transition temperature (*T*_g_); as water molecules plasticize the material, the *T*_g_ decreases; and once below that of the ambient temperature, shape recovery will take place.^55−58^ Since cellulose nanofibrils are dominated by extended chains with long-range order, the present mechanism is different and related to elastic deformation of highly flexible fibrils of high axial modulus, in combination with moisture-sensitive and reversible interfibril adhesion.

### 2.5 Thermal Properties and Generality of Preparation Method

**Figure 7:**
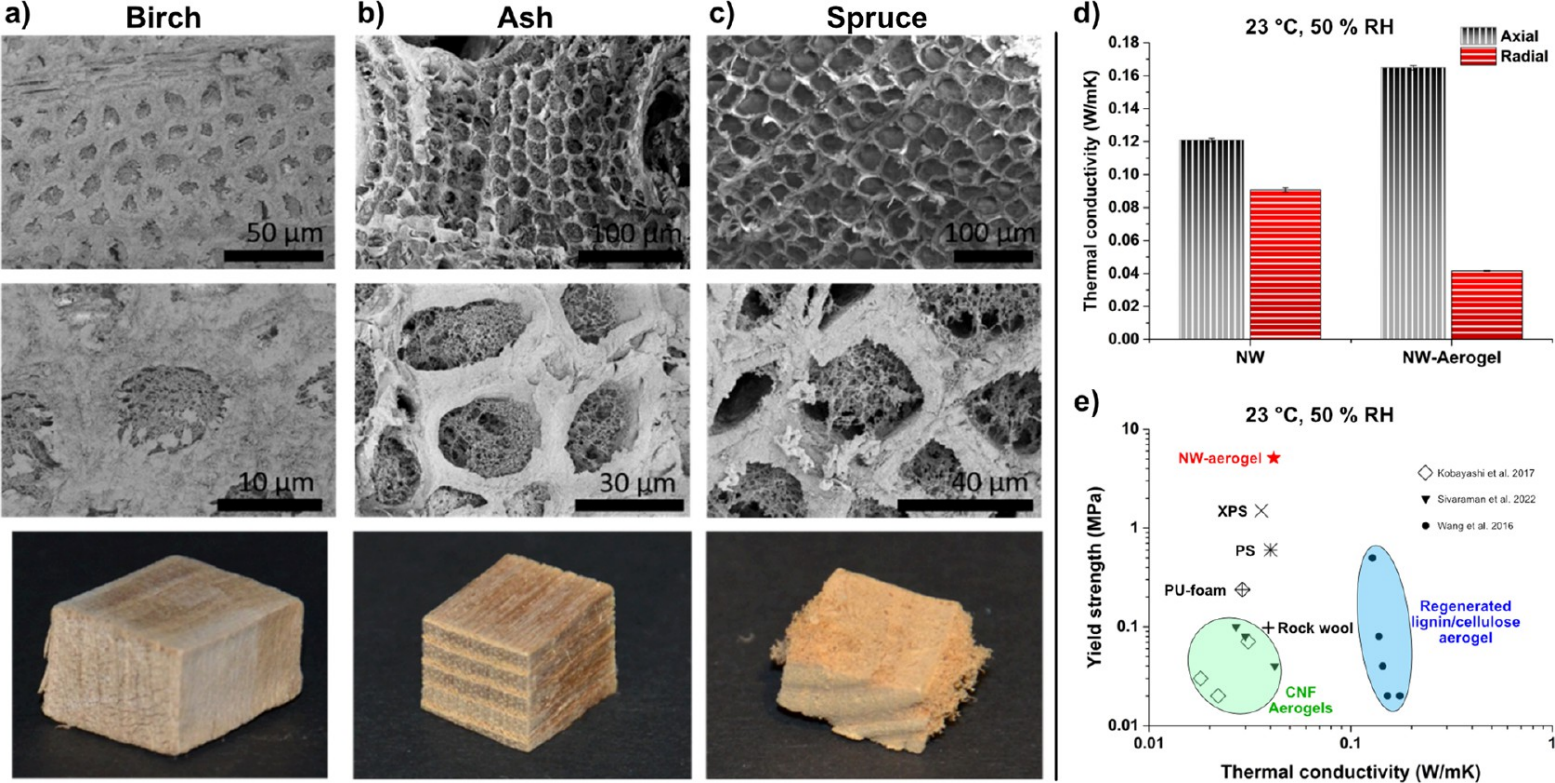
SEM images of (a) birch, (b) ash, and (c) spruce. First row: low magnification images of the morphology from cross-section cuts. Second row: higher magnification images of cell walls, focusing on the lumen nanofibril filling. Third row: digital photographs of each respective sample used for SEM analysis. (d) Thermal conductivity in radial and axial directions of NW and NW-Aerogel based on balsa and (e) comparison of yield strength and thermal conductivity to cellulosic aerogels,^59,60^ regenerated lignin/cellulose aerogel,^61^ and some commercially used thermal insulators.^62−64^ The NW-Aerogel was prepared *via* freeze-drying for thermal property measurements.

The preparation methodology developed for aerogel fabrication is scalable and could be extended to other wood species, including hardwood (birch, ash) and softwood (spruce) (Figure 7a–c). Wood aerogel structure differs depending on the wood species, densities, *etc.* As a diffuse-porous hardwood, balsa obtained well-defined networks as the cell walls became thinner due to the IL, leaving the middle lamella appearance intact. For birch (Figure 7a), dense network formation within the lumen was observed, which could be ascribed to diffusion of the organic IL electrolyte and thicker cell walls subjected to the dissolution. As a ring-porous hardwood, ash (Figure 7b) showed better preservation of sample dimensions with a less dense nanofibril network in the lumen and well-maintained cell walls. Softwood spruce did also form nanofibrillated networks within the lumen. The cell difference between earlywood and latewood results in fiber separation in the latewood region since dissolution kinetics is difficult to control when cell wall thicknesses are uneven (Figure 7c).

With its porous structure and good mechanical properties, the NW-Aerogel is a prospect for thermal insulation. Figure 7d shows the thermal conductivity of NW and NW-Aerogel; the specific heat capacity (*C*_*p*_) is seen in Figure S9. NW shows a radial thermal conductivity (λ_radial_) of 0.091 W/mK and axial thermal conductivity (λ_axial_) of 0.121 W/mK. NW-Aerogel showed a much lower λ_radial_ of 0.042 W/mK. This is mainly due to the improved nanoporosity of the cell walls and the fibrillated networks in the lumen. Figure 7e compares the λ_radial_ of NW-Aerogel to those of commercial insulation materials (polystyrene (PS), extruded polystyrene (XPS), polyurethane (PU) foam, and Rockwool),^62−64^ cellulosic aerogels,^59,60^ and regenerated cellulose/lignin aerogel.^61^ The all-wood NW-Aerogel shows great strength at the given λ value. λ_axial_ of NW-Aerogel is ∼0.16 W/mK, which is higher than that of NW and previously reported delignified wood aerogel.^22^ This could be ascribed to the increased density, high cellulose orientation of preserved cell walls, and possibly retention of lignin in the structure for better connectivity. A comparison of λ_radial_ and λ_axial_ to wood-based insulators of the literature is seen in Table S2 of the Supporting Information. The thermal insulation anisotropy (λ_axial_/λ_radial_) was ≈4, similar to high performance anisotropic thermal insulators in the literature.^12,65^ NW-Aerogel is exceptional due to the combination of low thermal conductivity with high strength and shape-memory function. The distinct structure–property relationships achieved after facile processing of wood for controlled nanostructure are of great scientific interest; the aerogels can also serve as substrates for advanced functional materials.

## 3 Conclusions

A strong all-wood aerogel containing lignin with shape-memory behavior was designed by wood nanostructure reassembly *via* partial dissolution and regeneration of the cell wall using a one-step IL ([MTBD]^+^[MMP]^−^/DMSO) treatment. Cell wall biopolymer composition was preserved while a fraction of cellulose and hemicellulose carbohydrates diffused out from the cell wall and formed nanofibril networks in the microscale lumen pores. The cell wall was highly swollen in the wet state, generating a large amount of nanoscale pores in the dry aerogels. The integrated wood aerogel structure leads to a high surface area (up to 220 m^2^/g) and low radial thermal conductivity (0.042 W/mK). Lignin was largely preserved in the structure, leading to excellent mechanical properties in the same range as native wood. The aerogel is shapeable, allowing for on-demand geometries, including folding and twisting in all directions of wood. Shape-memory behavior was apparent under stimuli of water from shaped dry aerogels for multiple cycles. The fabrication method could be applied to a variety of wood species, including balsa, birch, spruce, and ash. The methodology developed for high nanostructural control of lignin-containing wood aerogel can significantly contribute toward the design of advanced wood-based materials.

## 4 Experimental Section

### 4.1 Materials

Balsa wood (Ochroma pyramidale) of density 113 ± 3 kg/m^3^ was bought from Material AB, Sweden. Birch wood (*Betula pendula*, 558 ± 33 kg/m^3^), spruce wood (*Picea abies*, 407 ± 8 kg/m^3^), and ash wood (*Fraxinus excelsior*, 644 ± 15 kg/m^3^) were all bought from Calexico Wood AB. All wood types were cut to dimensions of 15 × 15 × 10 mm^3^ (tangential × radial × axial). Dimethyl sulfoxide ((CH_3_)_2_SO) was purchased from Sigma-Aldrich, Sweden. 7-Methyl-1,5,7-triazabicyclo[4.4.0]dec-5-ene (MTBD) was obtained from Liuotin Group OY. Dimethyl methylphosphonate (DMMP) (+99%) was purchased from Merck or ABCR GmbH. Chemicals used for lignin analysis were as follows; 1,4-dioxane, pyridine, acetic anhydride, methanol, and toluene (all purchased from Sigma-Aldrich, Sweden)

#### Ionic Liquid Preparation

Distilled MTBD (613 g, 3.6 mol, 1.2 equiv) was mixed with dimethyl methylphosphonate (DMMP) (384 g, 3 mol, 1.0 equiv) using a 2 L two-necked round-bottom flask. Subsequently, the flask was evacuated and backfilled with Argon. After that, the mixture was allowed to rest at room temperature (RT) for 10 min to dissipate the mild exothermic reaction observed and heated at 110 °C with vigorous stirring. Finally, the mixture was cooled to RT. The obtained ionic liquid had a dark yellow color.

### 4.2 NW-Aerogel Preparation

Wood aerogels were prepared by immersing native wood (NW) in DMSO until entirely soaked, followed by transferring NW to the organic electrolyte mixture [MTBD]^+^[MMP]^−^: DMSO, 20:80 wt %, in which it was treated for 48 h at 85 °C. The gel substrate was regenerated through the addition of Milli-Q water to the solution. Thereafter, *tert*-butanol was added to the rinsed samples (40:60 wt % *tert*-butanol:water) to minimize ice crystal growth during freezing. Samples were dried by rapid freezing in liquid nitrogen (−196 °C) followed by freeze-drying (−90 °C) for 2 days. These samples were first solvent exchanged from water to ethanol, followed by CPD in an Autosamdri-815.

### 4.3 Characterization

The sample morphology was studied using a field emission scanning electron microscope (SEM, Hitachi S-4800, Japan). All samples were sputtered (Cressington 208HR, U.K.) with a platinum/palladium layer for 20 s, giving a layer thickness of about 3 nm before the SEM analysis. Apparent density and porosity were assessed from oven-dried samples based on overnight heating at 105 °C and 30 min vacuum prior to measurements. Dimensions were measured using a caliper (Mitutoyo, digital caliper, Japan). The porosity was calculated using eq 1. Solid density of wood was assigned 1500 kg/m^3^.^66^
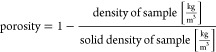
1The extractive content in wood was assessed gravimetrically after extraction with acetone for 3 h in a Soxhlet apparatus, and the ash content was determined according to the standard method ASTM D1102. Carbohydrate analysis was performed by grinding the samples in a Wiley mill (40 μm particles), followed by hydrolysis of the ground material using 37% H_2_SO_4_. The hydrolyzed constituents were introduced to a Dionex ICS-300 ion chromatography system (Thermo Fisher Scientific Inc., USA). Monosaccharides determined were arabinose, galactose, xylose, mannose, and glucose, in which cellulose content was assumed equivalent to the glucose content. Lignin content was analyzed with the Klason lignin method, according to the standard method TAPPI T 222 om-2.

Atomic force microscopy (AFM) measurements were performed on lyophilized microsection (cryo-microtomed), cleaned with nitrogen gas, and placed on a three-dimensional-printed sample holder below the microscope. AFM measurements were performed using the same microscope in the intermittent contact (AC) mode. Experimental conditions were as follows: silicon cantilever; tetrahedral silicon tip (spring constant (*k*) = 2.8 N/m, α = 25°, radius ∼ 10 nm) (ArrowTM, Nanoworld, Switzerland); scan area = 512 × 512 sample points; set point = 0.5 V; P-gain, 5%; I-gain, 5%; driving amplitude, 0.050 Vpp; driving frequency, 83750 Hz. The measurements were carried out in the dry state of the blocks, and the cantilever was placed under a 20× objective (Carl Zeiss, Germany).

Nitrogen physisorption was performed to assess the specific surface area and pore-size distribution. Samples of about 1 g were outgassed at 80 °C for 1 day prior to the physisorption analysis. The analysis was performed with a Micromeritics ASAP 2020 between the relative pressures of 0.05 and 1.0 *P*/*P*_o_ under liquid nitrogen conditions of −196 °C. The BET-specific surface area (SSA) was assessed by analyzing the adsorption isotherm between the relative pressures 0.05 and 0.25 utilizing the multipoint Brunauer–Emmet–Teller (BET) theory. The desorption isotherm was analyzed to study pore-size distribution (PSD) and pore volume according to the Barret–Joyner–Halenda (BJH) model.^67^

Cryo-microtomy, Raman imaging, and histochemical staining were performed to probe the microchemistry. The samples were frozen on an aluminum platform in liquid nitrogen (−196 °C) for at least 2 min. Thereafter, the frozen blocks were used to cut thin microsections (∼10 μm) using a cryo-microtome (CM 3050 S) from Leica (Biosystems Nussloch GmbH, Germany). For Raman imaging, the cross-sections were placed with a drop of distilled water on a glass slide, covered with a glass coverslip (0.17 mm thick), and sealed with nail polish. The embedded microsections were put on a piezomotorized scan stage (*x*–*y*) to be scanned in 300 nm steps with a confocal Raman microscope (CRM) (alpha 300 RA, WITec GmbH, Germany). A linear polarized (0°) coherent compass sapphire green laser (λ = 532 nm; laser power = 40 mW; WITec, Germany) was focused through a 100× oil immersion objective (NA, 1.4; Carl Zeiss, Jena, Germany) onto the microsection and the Raman scattering signal collected back through the same objective, passing a band-pass filter before guided *via* an optic multifiber (i.d. = 50 μm) to the spectrometer (600 g mm^–1^ grating; UHTS 300 WITec, Germany) and the attached CCD camera (Andor DU401ABV, Belfast, North Ireland). At every pixel, one full wavenumber spectrum was acquired with an integration time between 0.05 and 0.1 s. The cell wall polymers were scanned with a 330 nm spatial resolution The control (WITec) acquisition software was used to set experimental parameters for hyperspectral image acquisition and the WITec Project FIVE Plus software for cosmic ray removal, background subtractions, and “true component analysis”. The derived true component spectra were further analyzed using a mixture analysis algorithm (orthogonal matching pursuit)^68^ and a reference library to conclude the composition of the “components” found in native wood and the aerogel. For the histochemical staining, microsections were stained with Fuchsin–Chrysoidine–Astrablue (FCA) solution (0.1 mg/mL of Fuchsin, 0.143 mg/mL Chrysoidine, 1.25 mg/mL Astra blue and acetic acid (v:v = 1:50)) and then washed step by step with ethanol (30%, 70%) and isopropanol before being embedded and imaged using a Labophot-2 microscope (Nikon Corp., Tokyo, Japan).

To study the lignin structure *via* NMR, debarked birch wood was milled using a Wiley mini-mill 3383-L70 (Thomas Scientific, USA) through a 20-mesh. The wood powder was further milled in a Retsch PM400 apparatus (Ninolab, Sweden) for 24 h using 1 h milling intervals and 30 min pause. The grinding jar was purged with nitrogen before milling. For milled wood lignin (MWL) extraction, the Björkman protocol was used with some modifications.^69^ Ball milled birch (3 g) were mixed with 96% (v:v) 1,4-dioxane (75 mL) for 72 h at RT. The extract was collected by centrifugation, and the dioxane was completely removed under reduced pressure in a rotary evaporator. The extracted MWL was then treated according to the partial dissolution/regeneration protocol of aerogel preparation using [MTBD]^+^[MMP]^−^/DMSO-*d*_6_ for 48 h at 85 °C. The lignin was then precipitated in Milli-Q water and washed four times with Milli-Q water before freeze-drying. To study the lignin, acetylation was needed following the protocol by Gellerstedt with some modifications as reported in previous work.^70,71^ In short, 2 mg of the material was mixed with 100 μL of pyridine and acetic anhydride in a 1:1 ratio overnight at RT. The solution was dried under nitrogen flow. Pyridine was efficiently removed by the addition of a cold methanol and toluene solution in a 1:1 ratio. The following NMR measurements were performed in a Bruker 400 DMX instrument (Bruker Corp,, Billerica, MA, USA) with a multinuclear inverse Z-grad probe. Heteronuclear single-quantum-coherence (HSQC) experiments were performed with the pulse sequence hsqcetgpsi. The pulse length was optimized at 9.2 s, and a relaxation delay of 1.49 s was used. The number of scans was set to 176. Thirty mg of sample were dissolved in 550 μL *d*_6_-DMSO for the experiment. The spectra were processed with MestreNova (Mestrelab Research).

Further chemical analysis was performed using attenuated total reflectance Fourier transform infrared spectroscopy (ATR-FTIR) and solid-state cross-polarization magic angle spinning (CP-MAS) ^13^C nuclear magnetic resonance (NMR). ATR-FTIR was performed using a PerkinElmer spectrum 100 FTIR integrated with an ATR unit of diamond crystal (Graseby Specac Ltd., U.K.). All samples were measured at RT between the range 4000 and 600 cm^–1^ and each sample was scanned 8 times. NMR was used to quantify the cellulose crystallinity index (CI) and was analyzed on a Bruker Avance II AQS 400 SB running at 9.4 T, equipped with a double air-bearing probe of 4 mm. The CI was obtained by deconvolution of the C4 signal using a nonlinear least-squares fitting.

Cellulose crystal changes and degree of cellulose microfibrils orientation were studied using wide-angle X-ray scattering (WAXS). Transmission tests were performed on a SAXSpoint 2.0 system (Cu Kα, λ = 1.5418 Å; Anton Paar, Austria) with an Eiger R1M detector (Dectris Ltd., Switzerland) of pixel size 75 × 75 μm^2^ and a beam size of ≈500 μm. The sample to detector distance (SSD) was 111 mm as thin (≈1 mm) wet samples were mounted perpendicular to the incident beam with the wood fiber direction in a vertical position. The sample holder was thereafter sealed with a Kapton film. The obtained 2-D patterns were analyzed with the software SAXSanalysis in which they were transformed into 1-D diffractograms, including background subtraction of the Kapton film and water signal. Azimuthal integration was performed on the Debye–Scherrer ring of the (200) crystal plane. Full width at half-maximum (fwhm) could be analyzed through a Gaussian fit of the obtained intensity profiles, and eq 2 was used to calculate the orientation factor (*f*). For SAXS measurements, wet samples were measured on the same system as WAXS, using an SSD of 562 mm.
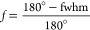
2Mechanical properties were evaluated by three-point bending, tensile, and compression. Cyclic three-point bending was executed on an Instron 5944 with a 500 N load cell using a strain rate of 6 mm/min. Before testing, all specimens were preconditioned at 23 °C and 50% RH for at least 2 days. Tensile tests wet samples were performed using an Instron 5944 using a 500 N load cell. The tests proceeded with a strain rate of 3 mm/min, using a gauge length of 25 mm, where the samples had a thickness of 2 mm and a width of 4 mm. All samples were kept in Milli-Q water for at least 1 week prior to testing. Longitudinal and radial compression tests were performed using an Instron 5966 having a load cell of 10 kN with a strain rate of 10%/min. All mechanical tests were performed in a controlled room of 23 °C and 50% relative humidity, in which the samples were conditioned for at least 2 days before testing. Tested samples had similar dimensions of 10 × 15 × 15 mm^3^ (longitudinal × radial × tangential).

Mirror-assisted multiview digital image correlation (MV-DIC) capable of obtaining panoramic surface strain distribution was used to measure the inhomogeneous and complicated deformation behavior of native wood (NW) and the NW-Aerogel. Digital image correlation (DIC)^72^ is an optical and noncontact method for full field surface deformation measurements. The measurement setup can be seen in Figure S10. NW and NW-Aerogel with a nominal dimension of 10 × 5 × 5 mm^3^ (longitudinal × radial × tangential) were compressed with preset constant strain rate of 10%/min by an Instron E1000 compression machine equipped with a 10 kN load cell. The image series recorded during the compression progress were processed to retrieve the shape and strain evolution on the whole surface (expect the two surfaces contact with the compression head) of the samples. Detailed information regarding the setup and procedure can be found in the Supporting Information.

Thermal conductivity of the NW-Aerogel was characterized using an anisotropic setting on a TPS 2500 S thermal constants analyzer (Hot Disk, Sweden). The characterization was performed according to ISO 22007-2 (Part 2: Transient plane heat source (hot disc) method) standard. The sensor model 5465 (radius, 3.2 mm) was clamped between two samples and lightly compressed with a steel rod to ensure complete contact with the sensor. Freeze-dried aerogels of 20 × 20 × 10 mm^3^ (radial × tangential × longitudinal) were used, and the analysis was carried out using a heating of 15 mW under 5 s for each respective sample. Between measurements, a 10 min delay transpired and each sample was measured at least six consecutive times. All measurements were performed under RT of 23 °C and 50% relative humidity (RH). Prior to analysis, all samples were conditioned at the same condition of 23 °C and 50% RH for more than 2 days. The anisotropic mode requires specific heat capacities (*C*_*p*_), which were measured using DSC (Mettler Toledo DSC1, Switzerland). A sapphire standard was utilized for accurate *C*_*p*_ values, and a predrying protocol (30 min, 105 °C) in the DSC was programmed to ensure dry specimen. The dynamic analysis followed the drying from −20 to 50 °C under N_2_ atmosphere (10 °C/min).

## Supporting Information Available

Wood treatment mass balance; pore-size distribution; ambient drying and reswelling; larger area confocal Raman images; confocal Raman images of thin-out-diffused cell wall; large area FCA stained samples; heteronuclear single quantum coherence; Kratky plots; three-point bending test; specific heat capacities; literature comparisons of thermal properties and material yield; illustration of mirror-assisted MV-DIC setup and measurement (PDF)
Video S1 showing cell wall dissolution with interface between fibril network and cell wall (MP4)
Video S2 showing NW-aerogel shape memory with water stimulus (MP4)
Video S3 showing NW strain field reaction (MP4)
Video S4 showing NW strain field reaction (MP4)
Video S5 showing panoramic surface deformation of NW (MP4)
Video S6 showing panoramic surface deformation of NW (MP4)

## References

[ref1] Habibi Y.; Lucia L. A.; Rojas O. J. Cellulose Nanocrystals: Chemistry, Self-Assembly, and Applications. Chem. Rev. 2010, 110, 3479–3500. 10.1021/cr900339w. 20201500

[ref2] French A. D.; Bertoniere N. R.; Brown R. M.; Chanzy H.; Gray D.; Hattori K.; Glasser W. Cellulose. In Kirk-Othmer Encyclopedia of Chemical Technology; John Wiley & Sons: New York, 2003. 10.1002/0471238961.0305121206180514.a01.pub2.

[ref3] Sakurada I.; Nukushina Y.; Ito T. Experimental Determination of the Elastic Modulus of Crystalline Regions in Oriented Polymers. J. Polym. Sci. 1962, 57, 651–660. 10.1002/pol.1962.1205716551.

[ref4] Kim C. H.; Youn H. J.; Lee H. L. Preparation of Cross-Linked Cellulose Nanofibril Aerogel with Water Absorbency and Shape Recovery. Cellulose 2015, 22, 3715–3724. 10.1007/s10570-015-0745-5.

[ref5] Xiao X.; Huang X.; Wang A.; Cao S.; Noroozi M.; Panahi-Sarmad M. Subtle Devising of Electro-Induced Shape Memory Behavior for Cellulose/Graphene Aerogel Nanocomposite. Carbohydr. Polym. 2022, 281, 119042 10.1016/j.carbpol.2021.119042. 35074116

[ref6] Jiang F.; Hsieh Y.-l. Super Water Absorbing and Shape Memory Nanocellulose Aerogels from TEMPO-Oxidized Cellulose Nanofibrils *via* Cyclic Freezing-Thawing. J. Mater. Chem. A 2014, 2, 350–359. 10.1039/C3TA13629A.

[ref7] Gu J.; Hu C.; Zhang W.; Dichiara A. B. Reagentless Preparation of Shape Memory Cellulose Nanofibril Aerogels Decorated with Pd Nanoparticles and Their Application in Dye Discoloration. Appl. Catal., B 2018, 237, 482–490. 10.1016/j.apcatb.2018.06.002.

[ref8] Zhang W.; Zhang Y.; Lu C.; Deng Y. Aerogels from Crosslinked Cellulose Nano/Micro-Fibrils and Their Fast Shape Recovery Property in Water. J. Mater. Chem. 2012, 22, 11642–11650. 10.1039/c2jm30688c.

[ref9] Li J.; Zuo K.; Wu W.; Xu Z.; Yi Y.; Jing Y.; Dai H.; Fang G. Shape Memory Aerogels from Nanocellulose and Polyethyleneimine As a Novel Adsorbent for Removal of Cu(II) and Pb(II). Carbohydr. Polym. 2018, 196, 376–384. 10.1016/j.carbpol.2018.05.015. 29891309

[ref10] Özparpucu M.; Gierlinger N.; Cesarino I.; Burgert I.; Boerjan W.; Rüggeberg M. Significant Influence of Lignin on Axial Elastic Modulus of Poplar Wood at Low Microfibril Angles under Wet Conditions. J. Exp. Bot. 2019, 70, 4039–4047. 10.1093/jxb/erz180. 31187131 PMC6685656

[ref11] Song J.; Chen C.; Yang Z.; Kuang Y.; Li T.; Li Y.; Huang H.; Kierzewski I.; Liu B.; He S.; Gao T.; Yuruker S. U.; Gong A.; Yang B.; Hu L. Highly Compressible, Anisotropic Aerogel with Aligned Cellulose Nanofibers. ACS Nano 2018, 12, 140–147. 10.1021/acsnano.7b04246. 29257663

[ref12] Sun H.; Bi H.; Lin X.; Cai L.; Xu M. Lightweight, Anisotropic, Compressible, and Thermally-Insulating Wood Aerogels with Aligned Cellulose Fibers. Polymers 2020, 12, 165 10.3390/polym12010165. 31936375 PMC7022930

[ref13] Li T.; Song J.; Zhao X.; Yang Z.; Pastel G.; Xu S.; Jia C.; Dai J.; Chen C.; Gong A.; Jiang F.; Yao Y.; Fan T.; Yang B.; Wågberg L.; Yang R.; Hu L. Anisotropic, Lightweight, Strong, and Super Thermally Insulating Nanowood with Naturally Aligned Nanocellulose. Sci. Adv. 2018, 4, 10.1126/sciadv.aar3724. PMC5844708 29536048

[ref14] Zhao Y.; You D.; Yang W.; Yu H.; Pan Q.; Song S. Cobalt Nanoparticle–Carbon Nanoplate As the Solar Absorber of a Wood Aerogel Evaporator for Continuously Efficient Desalination. Environ. Sci.: Water Res. Technol. 2021, 8, 151–161. 10.1039/D1EW00593F.

[ref15] Zhang Q.; Li L.; Jiang B.; Zhang H.; He N.; Yang S.; Tang D.; Song Y. Flexible and Mildew-Resistant Wood-Derived Aerogel for Stable and Efficient Solar Desalination. ACS Appl. Mater. Interfaces 2020, 12, 28179–28187. 10.1021/acsami.0c05806. 32489094

[ref16] Wang K.; Liu X.; Tan Y.; Zhang W.; Zhang S.; Li J. Two-Dimensional Membrane and Three-Dimensional Bulk Aerogel Materials via Top-Down Wood Nanotechnology for Multibehavioral and Reusable Oil/Water Separation. Chem. Eng. J. 2019, 371, 769–780. 10.1016/j.cej.2019.04.108.

[ref17] Kong W.; Wang C.; Jia C.; Kuang Y.; Pastel G.; Chen C.; Chen G.; He S.; Huang H.; Zhang J.; Wang S.; Hu L. Muscle-Inspired Highly Anisotropic, Strong, Ion-Conductive Hydrogels. Adv. Mater. 2018, 30, 1801934 10.1002/adma.201801934. 30101467

[ref18] Chen C. C.; Wang Y. R.; Wu Q. J.; Wan Z. M.; Li D. G.; Jin Y. C. Highly Strong and Flexible Composite Hydrogel Reinforced by Aligned Wood Cellulose Skeleton via Alkali Treatment for Muscle-Like Sensors. Chem. Eng. J. 2020, 400, 125876 10.1016/j.cej.2020.125876.

[ref19] Wang S.; Chen H.; Li K.; Koskela S.; Berglund L. A.; Zhou Q. Strong, Transparent, and Thermochromic Composite Hydrogel from Wood Derived Highly Mesoporous Cellulose Network and PNIPAM. Composites, Part A 2022, 154, 106757 10.1016/j.compositesa.2021.106757.

[ref20] Sjöström E. The Structure of Wood. In Wood Chemistry, 2nd ed.; Sjöström E., Ed.; Academic Press: San Diego, CA, USA, 1993; Chapter 1, pp 1–20. 10.1016/B978-0-08-092589-9.50005-X.

[ref21] Garemark J.; Yang X.; Sheng X.; Cheung O.; Sun L.; Berglund L. A.; Li Y. Top-Down Approach Making Anisotropic Cellulose Aerogels As Universal Substrates for Multifunctionalization. ACS Nano 2020, 14, 7111–7120. 10.1021/acsnano.0c01888. 32413254 PMC7497664

[ref22] Garemark J.; Perea-Buceta J. E.; Rico Del Cerro D.; Hall S.; Berke B.; Kilpelainen I.; Berglund L. A.; Li Y. Nanostructurally Controllable Strong Wood Aerogel toward Efficient Thermal Insulation. ACS Appl. Mater. Interfaces 2022, 14, 24697–24707. 10.1021/acsami.2c04584. 35511115 PMC9164199

[ref23] Li Y.; Cui J.; Shen H.; Liu C.; Wu P.; Qian Z.; Duan Y.; Liu D. Useful Spontaneous Hygroelectricity from Ambient Air by Ionic Wood. Nano Energy 2022, 96, 107065 10.1016/j.nanoen.2022.107065.

[ref24] Jin H.; Nishiyama Y.; Wada M.; Kuga S. Nanofibrillar Cellulose Aerogels. Colloids Surf., A 2004, 240, 63–67. 10.1016/j.colsurfa.2004.03.007.

[ref25] Ren Z.; Zarepoor M.; Huang X.; Sabelhaus A. P.; Majidi C. Shape Memory Alloy (SMA) Actuator With Embedded Liquid Metal Curvature Sensor for Closed-Loop Control. Front. Robot. AI 2021, 8, 8 10.3389/frobt.2021.599650. PMC8059551 33898528

[ref26] Li L.; Shi P.; Hua L.; An J.; Gong Y.; Chen R.; Yu C.; Hua W.; Xiu F.; Zhou J.; Gao G.; Jin Z.; Sun G.; Huang W. Design of a Wearable and Shape-Memory Fibriform Sensor for the Detection of Multimodal Deformation. Nanoscale 2018, 10, 118–123. 10.1039/C7NR06219B. 29211073

[ref27] Gierlinger N.; Keplinger T.; Harrington M. Imaging of Plant Cell Walls by Confocal Raman Microscopy. Nat. Protoc. 2012, 7, 1694–1708. 10.1038/nprot.2012.092. 22918387

[ref28] Kraus J. E.; de Sousa H. C.; Rezende M. H.; Castro N. M.; Vecchi C.; Luque R. Astra Blue and Basic Fuchsin Double Staining of Plant Materials. Biotechnol. Histochem. 1998, 73, 235–243. 10.3109/10520299809141117. 9829416

[ref29] Labbé N.; Rials T. G.; Kelley S. S.; Cheng Z.-M.; Kim J.-Y.; Li Y. FT-IR Imaging and Pyrolysis-Molecular Beam Mass Spectrometry: New Tools to Investigate Wood Tissues. Wood Sci. Technol. 2005, 39, 61–76. 10.1007/s00226-004-0274-0.

[ref30] Lehto J.; Louhelainen J.; Kłosińska T.; Drożdżek M.; Alén R. Characterization of Alkali-Extracted Wood by FTIR-ATR Spectroscopy. Biomass Convers. Biorefin. 2018, 8, 847–855. 10.1007/s13399-018-0327-5.

[ref31] Jiang Z.; Yi J.; Li J.; He T.; Hu C. Promoting Effect of Sodium Chloride on the Solubilization and Depolymerization of Cellulose from Raw Biomass Materials in Water. ChemSusChem 2015, 8, 1901–1907. 10.1002/cssc.201500158. 25916895

[ref32] Boukir A.; Fellak S.; Doumenq P. Structural Characterization of Argania Spinosa Moroccan Wooden Artifacts during Natural Degradation Progress Using Infrared Spectroscopy (ATR-FTIR) and X-Ray Diffraction (XRD). Heliyon 2019, 5, e02477 10.1016/j.heliyon.2019.e02477. 31687572 PMC6819844

[ref33] Ling Z.; Chen S.; Zhang X.; Takabe K.; Xu F. Unraveling Variations of Crystalline Cellulose Induced by Ionic Liquid and Their Effects on Enzymatic Hydrolysis. Sci. Rep. 2017, 7, 10230 10.1038/s41598-017-09885-9. 28860612 PMC5579251

[ref34] Shi J.; Xing D.; Lia J. FTIR Studies of the Changes in Wood Chemistry from Wood Forming Tissue under Inclined Treatment. Energy Procedia 2012, 16, 758–762. 10.1016/j.egypro.2012.01.122.

[ref35] Park S.; Baker J. O.; Himmel M. E.; Parilla P. A.; Johnson D. K. Cellulose Crystallinity Index: Measurement Techniques and Their Impact on Interpreting Cellulase Performance. Biotechnol. Biofuels 2010, 3, 10 10.1186/1754-6834-3-10. 20497524 PMC2890632

[ref36] Daicho K.; Fujisawa S.; Kobayashi K.; Saito T.; Ashida J. Cross-Polarization Dynamics and Conformational Study of Variously Sized Cellulose Crystallites Using Solid-State 13C NMR. J. Wood Sci. 2020, 66, 62 10.1186/s10086-020-01909-9.

[ref37] Ago M.; Endo T.; Hirotsu T. Crystalline Transformation of Native Cellulose from Cellulose I to Cellulose ID Polymorph by a Ball-Milling Method with a Specific Amount of Water. Cellulose 2004, 11, 163–167. 10.1023/B:CELL.0000025423.32330.fa.

[ref38] Idström A.; Schantz S.; Sundberg J.; Chmelka B. F.; Gatenholm P.; Nordstierna L. 13C NMR Assignments of Regenerated Cellulose from Solid-State 2D NMR Spectroscopy. Carbohydr. Polym. 2016, 151, 480–487. 10.1016/j.carbpol.2016.05.107. 27474592

[ref39] Gil A. M.; Neto C. P. Solid-State Nmr Studies Of Wood And Other Lignocellulosic Materials. In Annual Reports on NMR Spectroscopy, Vol. 37; Webb G. A., Ed.; Academic Press: San Diego, CA, USA, 1999; pp 75–117. 10.1016/S0066-4103(08)60014-9.

[ref40] Dalvi L. C.; Laine C.; Virtanen T.; Liitiä T.; Tenhunen T.-M.; Orelma H.; Tammelin T.; Tamminen T. Study of Xylan and Cellulose Interactions Monitored with Solid-State NMR and QCM-D. Holzforschung 2020, 74, 643–653. 10.1515/hf-2019-0221.

[ref41] Jakob H. F.; Tschegg S. E.; Fratzl P. Hydration Dependence of the Wood-Cell Wall Structure in Picea abies. A Small-Angle X-ray Scattering Study. Macromolecules 1996, 29, 8435–8440. 10.1021/ma9605661.

[ref42] Chen P.; Li Y.; Nishiyama Y.; Pingali S. V.; O’Neill H. M.; Zhang Q.; Berglund L. A. Small Angle Neutron Scattering Shows Nanoscale PMMA Distribution in Transparent Wood Biocomposites. Nano Lett. 2021, 21, 2883–2890. 10.1021/acs.nanolett.0c05038. 33734720 PMC8050824

[ref43] Penttilä P.; Rautkari L.; Österberg M.; Schweins R. Small-Angle Scattering Model for Efficient Characterization of Wood Nanostructure and Moisture Behaviour. J. Appl. Crystallogr. 2019, 52, 369–377. 10.1107/S1600576719002012. 30996716 PMC6448686

[ref44] Jakob H. F.; Fengel D.; Tschegg S. E.; Fratzl P. The Elementary Cellulose Fibril in Picea abies: Comparison of Transmission Electron Microscopy, Small-Angle X-ray Scattering, and Wide-Angle X-ray Scattering Results. Macromolecules 1995, 28, 8782–8787. 10.1021/ma00130a010.

[ref45] Parviainen A.; King A. W. T.; Mutikainen I.; Hummel M.; Selg C.; Hauru L. K. J.; Sixta H.; Kilpeläinen I. Predicting Cellulose Solvating Capabilities of Acid–Base Conjugate Ionic Liquids. ChemSusChem 2013, 6, 2161–2169. 10.1002/cssc.201300143. 24106149

[ref46] Lindman B.; Karlström G.; Stigsson L. On the Mechanism of Dissolution of Cellulose. J. Mol. Liq. 2010, 156, 76–81. 10.1016/j.molliq.2010.04.016.

[ref47] El Seoud O. A.; Kostag M.; Jedvert K.; Malek N. I. Cellulose in Ionic Liquids and Alkaline Solutions: Advances in the Mechanisms of Biopolymer Dissolution and Regeneration. Polymers (Basel) 2019, 11, 1917 10.3390/polym11121917. 31766402 PMC6960809

[ref48] Gibson L. J.; Ashby M. F. Wood. In Cellular Solids: Structure and Properties, 2 ed.; Cambridge Solid State Science Series; Cambridge University Press: Cambridge, U.K., 1997; pp 387–428. 10.1017/CBO9781139878326.012.

[ref49] Chen B.; Pan B. Mirror-Assisted Multi-View Digital Image Correlation: Principles, Applications and Implementations. Opt. Lasers Eng. 2022, 149, 106786 10.1016/j.optlaseng.2021.106786.

[ref50] Poulsen J. S.; Moran P. M.; Shih C. F.; Byskov E. Kink Band Initiation and Band Broadening in Clear Wood under Compressive Loading. Mech. Mater. 1997, 25, 67–77. 10.1016/S0167-6636(96)00043-9.

[ref51] Safranski D. L. Introduction to Shape-Memory Polymers. In Shape-Memory Polymer Device Design; Safranski D. L., Griffis J. C., Eds.; William Andrew: Cambridge, U.K., 2017; Chapter 1, pp 1–22. 10.1016/B978-0-323-37797-3.00001-4.

[ref52] Lendlein A.; Kelch S. Shape-Memory Polymers. Angew. Chem., Int. Ed. 2002, 41, 2034–2057. 10.1002/1521-3773(20020617)41:12<2034::AID-ANIE2034>3.0.CO;2-M. 19746597

[ref53] Hanzon D. W.; Yu K.; Yakacki C. M. Activation Mechanisms of Shape-Memory Polymers. In Shape-Memory Polymer Device Design; Safranski D. L., Griffis J. C., Eds.; William Andrew: Cambridge, U.K., 2017; Chapter 5, pp 139–187. 10.1016/B978-0-323-37797-3.00005-1.

[ref54] Leire R.-R.; Leyre P.-Á.; Beñat A.; Juan M. G.-Z.; José Luis V. Shape Memory Hydrogels Based on Noncovalent Interactions. In Shape-Memory Materials; Ares A. E., Ed.; IntechOpen: Rijeka, Croatia, 2018; Chapter 6. 10.5772/intechopen.78013.

[ref55] Liu Y.; Li Y.; Chen H.; Yang G.; Zheng X.; Zhou S. Water-Induced Shape-Memory Poly(d,l-lactide)/Microcrystalline Cellulose Composites. Carbohydr. Polym. 2014, 104, 101–108. 10.1016/j.carbpol.2014.01.031. 24607166

[ref56] Leng J.; Lan X.; Liu Y.; Du S. Shape-Memory Polymers and Their Composites: Stimulus Methods and Applications. Prog. Mater. Sci. 2011, 56, 1077–1135. 10.1016/j.pmatsci.2011.03.001.

[ref57] Leng J.; Lv H.; Liu Y.; Du S. Water-Driven Programable Polyurethane Shape Memory Polymer: Demonstration and Mechanism. Appl. Phys. Lett. 2008, 92, 206105 10.1063/1.2936288.

[ref58] Lu H.; Liu Y.; Leng J.; Du S. Qualitative Separation of the Physical Swelling Effect on the Recovery Behavior of Shape Memory Polymer. Eur. Polym. J. 2010, 46, 1908–1914. 10.1016/j.eurpolymj.2010.06.013.

[ref59] Sivaraman D.; Siqueira G.; Maurya A. K.; Zhao S.; Koebel M. M.; Nyström G.; Lattuada M.; Malfait W. J. Superinsulating Nanocellulose Aerogels: Effect of Density and Nanofiber Alignment. Carbohydr. Polym. 2022, 292, 119675 10.1016/j.carbpol.2022.119675. 35725170

[ref60] Kobayashi Y.; Saito T.; Isogai A. Aerogels with 3D Ordered Nanofiber Skeletons of Liquid-Crystalline Nanocellulose Derivatives As Tough and Transparent Insulators. Angew. Chem., Int. Ed. 2014, 53, 10394–10397. 10.1002/anie.201405123. 24985785

[ref61] Wang C.; Xiong Y.; Fan B.; Yao Q.; Wang H.; Jin C.; Sun Q. Cellulose As an Adhesion Agent for the Synthesis of Lignin Aerogel with Strong Mechanical Performance, Sound-Absorption and Thermal Insulation. Sci. Rep. 2016, 6, 32383 10.1038/srep32383. 27562532 PMC5387396

[ref62] Marhoon I. I.; Rasheed A. K. Mechanical and Physical Properties of Glass Wool-Rigid Polyurethane Foam Composites. Al-Nahrain Univ. Coll. Eng. J. (NUCEJ) 2015, 18, 41–49.

[ref63] Buska A.; Mačiulaitis R. The Compressive Strength Properties of Mineral Wool Slabs: Influence of Structure Anisotropy and Methodical Factors. J. Civ. Eng. Manage. 2007, 13, 97–106. 10.3846/13923730.2007.9636425.

[ref64] Aksit M.; Zhao C.; Klose B.; Kreger K.; Schmidt H.-W.; Altstädt V. Extruded Polystyrene Foams with Enhanced Insulation and Mechanical Properties by a Benzene-Trisamide-Based Additive. Polymers 2019, 11, 268 10.3390/polym11020268. 30960252 PMC6419028

[ref65] Wicklein B.; Kocjan A.; Salazar-Alvarez G.; Carosio F.; Camino G.; Antonietti M.; Bergström L. Thermally Insulating and Fire-Retardant Lightweight Anisotropic Foams Based on Nanocellulose and Graphene Oxide. Nat. Nanotechnol. 2015, 10, 277–283. 10.1038/nnano.2014.248. 25362476

[ref66] Gibson L. J.; Ashby M. F. The Structure of Cellular Solids. In Cellular Solids: Structure and Properties, 2 ed.; Cambridge University Press: Cambridge, U.K., 1997; pp 15–51. 10.1017/CBO9781139878326.

[ref67] Brunauer S.; Emmett P. H.; Teller E. Adsorption of Gases in Multimolecular Layers. J. Am. Chem. Soc. 1938, 60, 309–319. 10.1021/ja01269a023.

[ref68] Pati Y. C.; Rezaiifar R.; Krishnaprasad P. S. Orthogonal Matching Pursuit: Recursive Function Approximation with Applications to Wavelet Decomposition. In Proceedings of 27th Asilomar Conference on Signals, Systems and Computers, Pacific Grove, CA, USA, Nov. 1–3, 1993; IEEE, 1993; Vol. 1, pp 40–44. 10.1109/ACSSC.1993.342465.

[ref69] Björkman A. Isolation of Lignin from Finely Divided Wood with Neutral Solvents. Nature 1954, 174, 1057–1058. 10.1038/1741057a0.

[ref70] Gellerstedt G. Gel Permeation Chromatography. In Methods in Lignin Chemistry; Lin S. Y., Dence C. W., Eds.; Springer Series in Wood Science; Springer: Berlin, 1992; pp 487–497. 10.1007/978-3-642-74065-7_34.

[ref71] Sapouna I.; Lawoko M. Deciphering Lignin Heterogeneity in Ball Milled Softwood: Unravelling the Synergy between the Supramolecular Cell Wall Structure and Molecular Events. Green Chem. 2021, 23, 3348–3364. 10.1039/D0GC04319B.

[ref72] Michael A M. A.; Orteu J.-J.; Schreier H. W. Digital Image Correlation (DIC). In Image Correlation for Shape, Motion and Deformation Measurements: Basic Concepts,Theory and Applications, Schreier H., Orteu J.-J., Sutton M. A., Eds.; Springer: Boston, 2009; pp 1–37. 10.1007/978-0-387-78747-3_5.

