# Supplementary material for: Strong, Shape-Memory Lignocellulosic Aerogel via Wood Cell Wall Nanoscale Reassembly
Source: ACS Nano. 2023 Jan 30;17(5):4775–89. doi: 10.1021/acsnano.2c11220 (PMC10018770; doi:10.1021/acsnano.2c11220)
Supplement: Supplementary file 1 — nn2c11220_si_001.pdf [file nn2c11220_si_001.pdf]

Supporting Information:

Strong, Shape-Memory Lignocellulosic Aerogel *via* Wood Cell Wall Nanoscale Reassembly

Jonas Garemark[1], Jesús E. Perea-Buceta[2], Martin Felhofer[3], Bin Chen[1], Maria F. Cortes Ruiz[1], Ioanna Sapouna[1,4], Notburga Gierlinger[3], Ilkka Antero Kilpeläinen[2], Lars A. Berglund[1], Yuanyuan Li[1]*

[1]Wallenberg Wood Science Center, Department of Fiber and Polymer Technology, KTH Royal Institute of Technology, SE-10044, Stockholm, Sweden

[2]Materials Chemistry Division, Department of Chemistry, Faculty of Science, University of Helsinki, 00560, Helsinki, Finland

[3]Department of Nanobiotechnology, Institute of Biophysics, University of Natural Resources and Life Sciences, 1190, Vienna, Austria

[4]Division of Glycoscience, Department of Chemistry, KTH Royal Institute of Technology, AlbaNova University Centre, 106 91, Stockholm, Sweden

*Corresponding author email: yua@kth.se

Key: Wood aerogel, cell wall reassembly, shape memory, strong

Table of contents:

1. NW-Aerogel supplementary results

Table S1. Mass balance of the wood treatment

Figure S1. BJH pore-size distribution of freeze-dried NW-Aerogel

Figure S2. Ambient dried and re-swelling of NW-Aerogel

Figure S3. Larger area confocal Raman images

Figure S4. Confocal Raman images of a thin out-diffused cell wall of NW-Aerogel

Figure S5. Large area FCA stained NW and NW-Aerogel

Figure S6. Heteronuclear single quantum coherence (HSQC) of treated milled wood lignin

Figure S7. Kratky plot of NW and NW-Aerogel

Figure S8. 3-point bending test of NW

Figure S9. Specific heat capacity (Cp) of NW and NW-Aerogel

Table S2. Literature comparison of thermal properties and material yield

2. DIC details

Figure S10. Illustration of mirror-assisted MV-DIC setup and measurement

Table S1. Mass balance of the wood treatment based on 100g of native wood (NW), including the wood components lost to the liquid stream

	Wood in	*Wood out*	
	**NW**	**NW-Aerogel**	***Wood lost in liquid stream***
**Cellulose** (g)	53.33	44.33 ± 0.35	9
**Hemicellulose** (g)	20.83	15.65 ± 0.85	5.18
**Lignin** (g)	22.25	15.96 ± 1.20	6.29
**Extractives** (g)	2.07 ± 0.51	0.63 ± 0.21	1.44
**Ash** (g)	1.52 ± 0.06	0.73 ± 0.16	0.79
Total (g)	100	77.3 ± 2.8	22.7

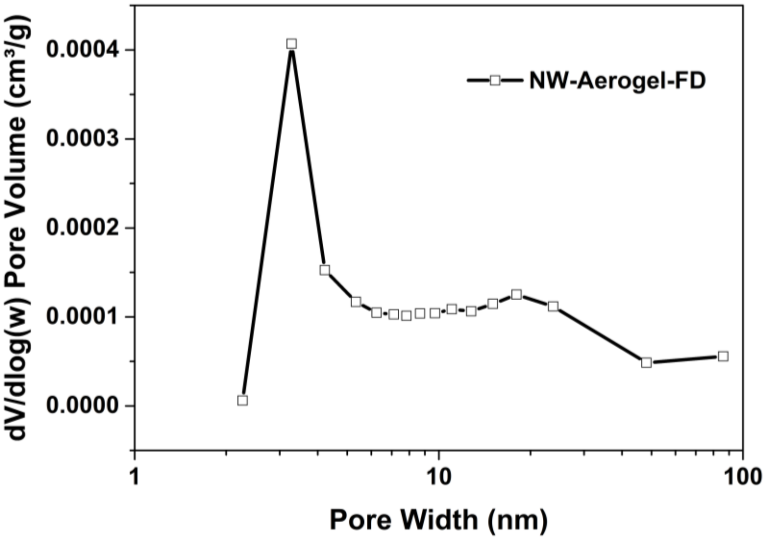

Figure S1. BJH pore-size distribution of freeze-dried NW-aerogel

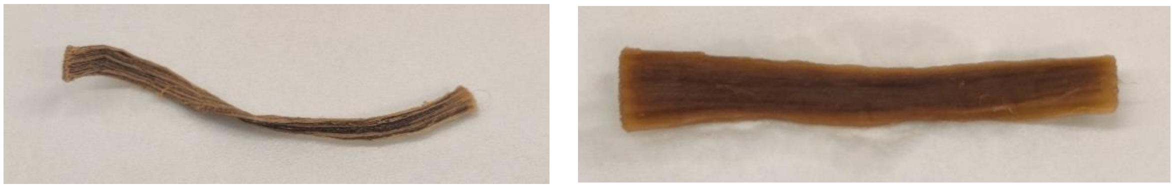

Figure S2. Left-hand side, ambient dried sample from water having a twisted shape. Right-hand side reswollen sample from water, returned back to its original flat shape

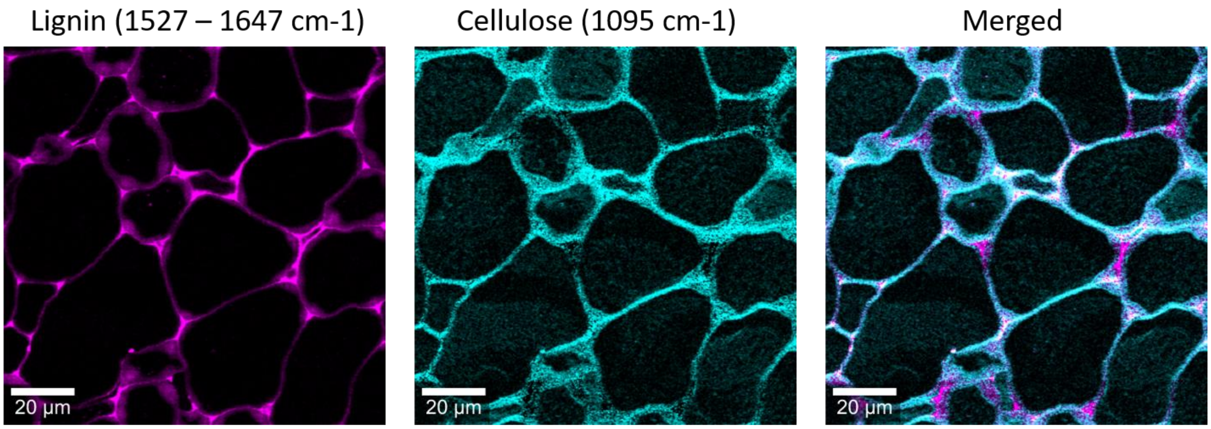

Figure S3. Larger area confocal Raman image. From the left, lignin signal is scanned and is clearly seen with intense signal form the CC and CML with decreasing signal towards the lumen. Middle image shows the cellulose signal, where the secondary cell walls show strongest signal followed by the fibrillated networks of the lumen space which is clearly seen. Furthest right is the combined image of lignin and cellulose signal.

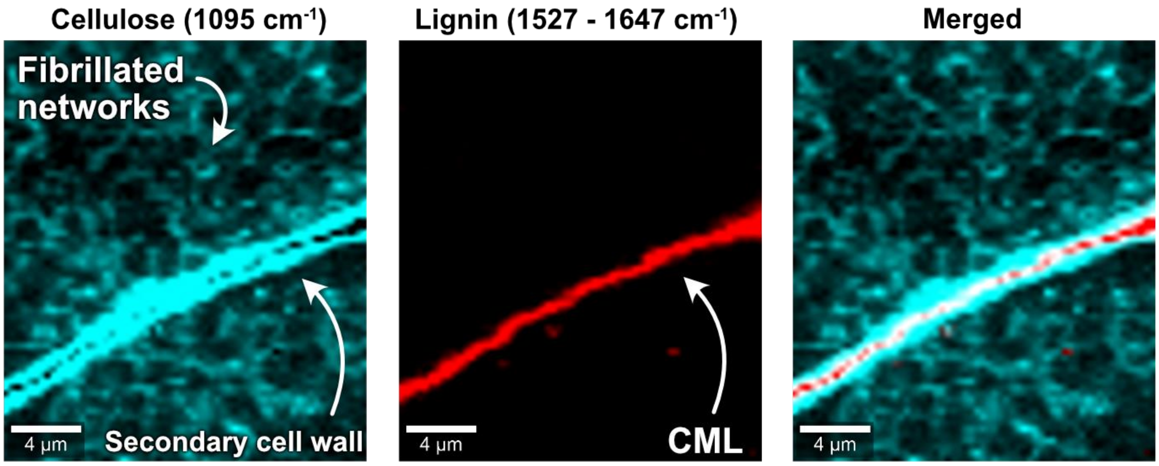

Figure S4. Confocal Raman images of a thin out-diffused cell wall. From the left, the cellulose signal is shown, clearly showing the strong cellulose signal from secondary cell wall and fibrillated networks within the lumen. It is apparent that the formed cellulose networks come from the cell walls. In the middle, lignin signal from the CML is shown, giving a notion that most of the remaining cell wall is from the lignin-rich CML. The right-hand image shows the merged signal which accentuates the message of cell walls mostly consisting of the lignin-rich CML after dissolution/regeneration of wood.

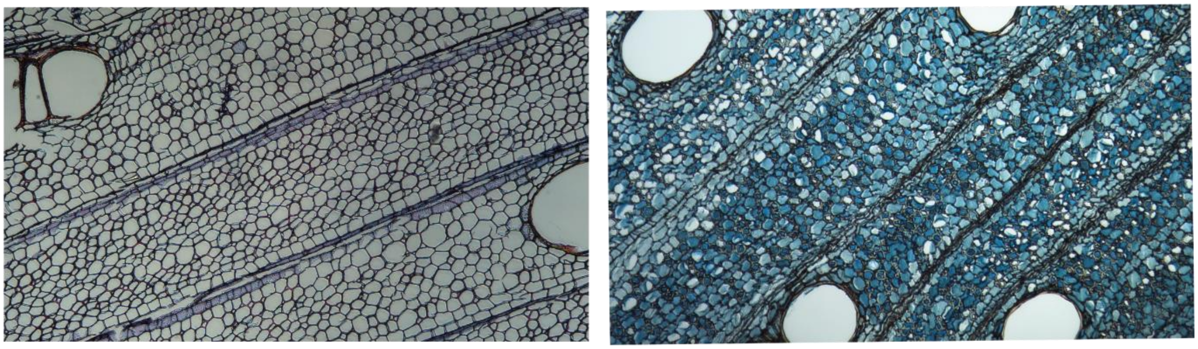
Figure S5. Left-hand side, native wood sample at 100x magnification and 10x. Right-hand side, NW-Aerogel sample at 100x and 10x using FCA staining. Blue represents carbohydrates and red lignin.

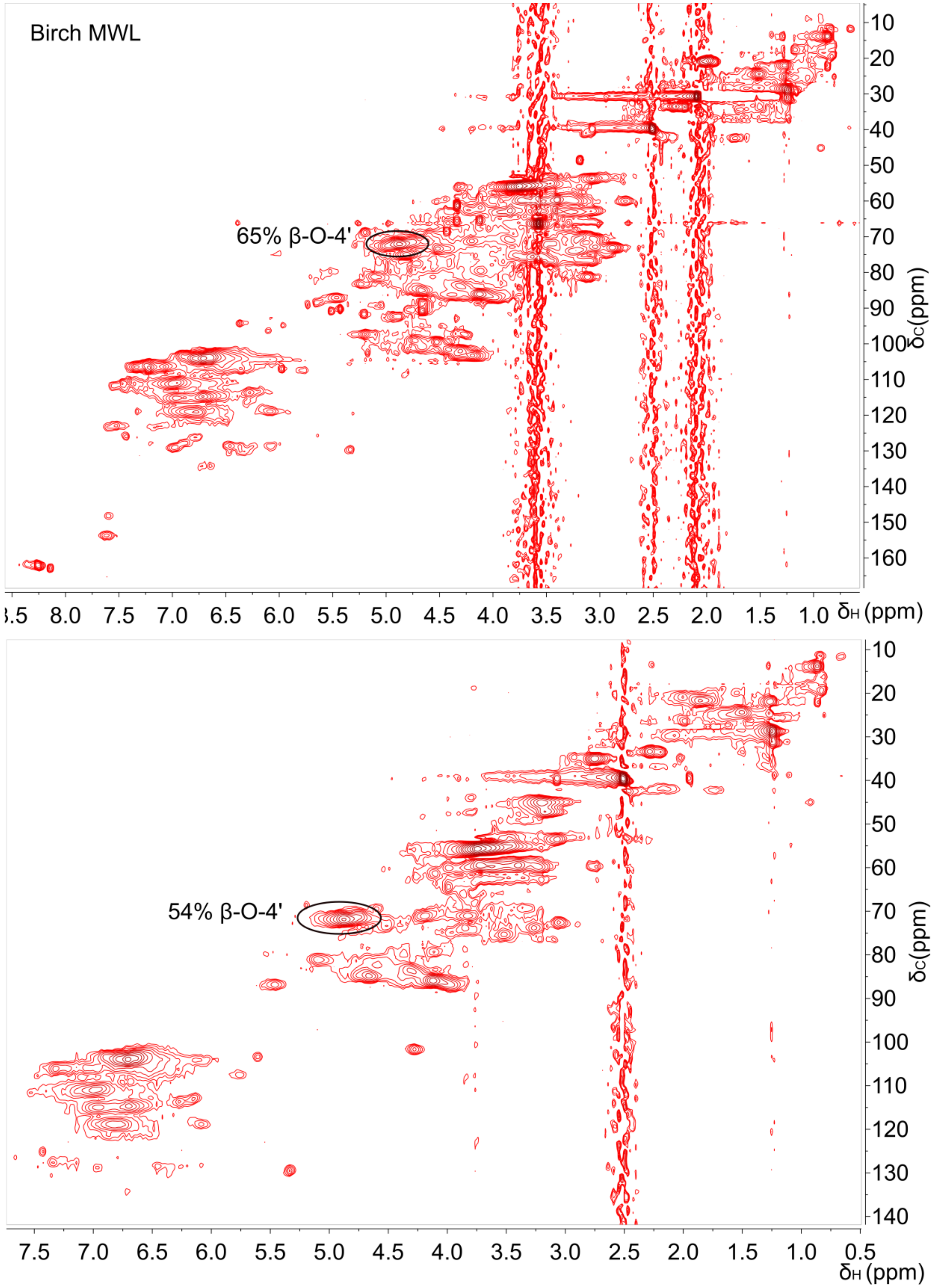

Figure S6. a) Heteronuclear single quantum coherence (HSQC) of milled wood lignin from birch compared to b) [MTBD][MMP]/DMSO treated milled wood lignin from birch through water precipitation.

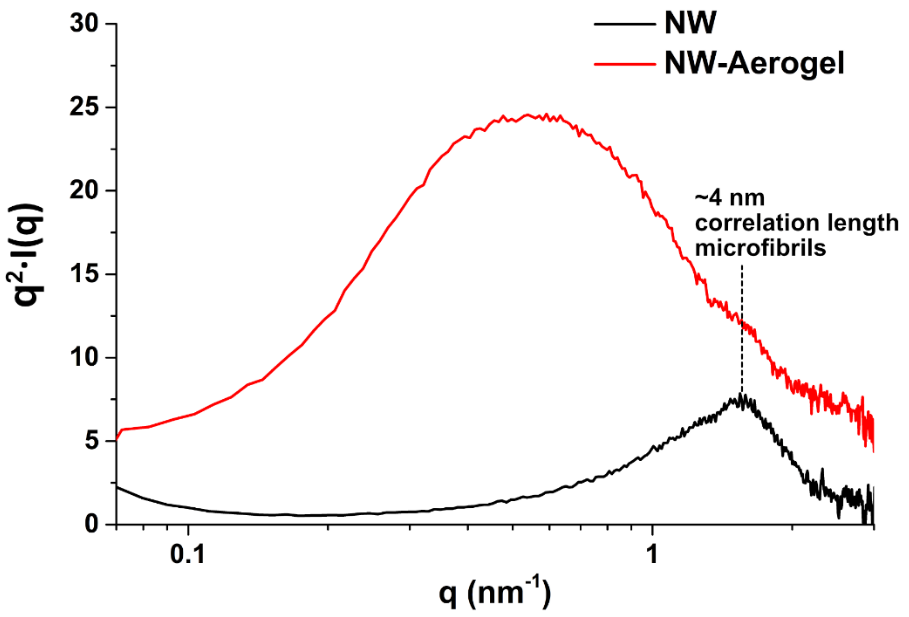

Figure S7. Kratky plot including NW and NW-Aerogel

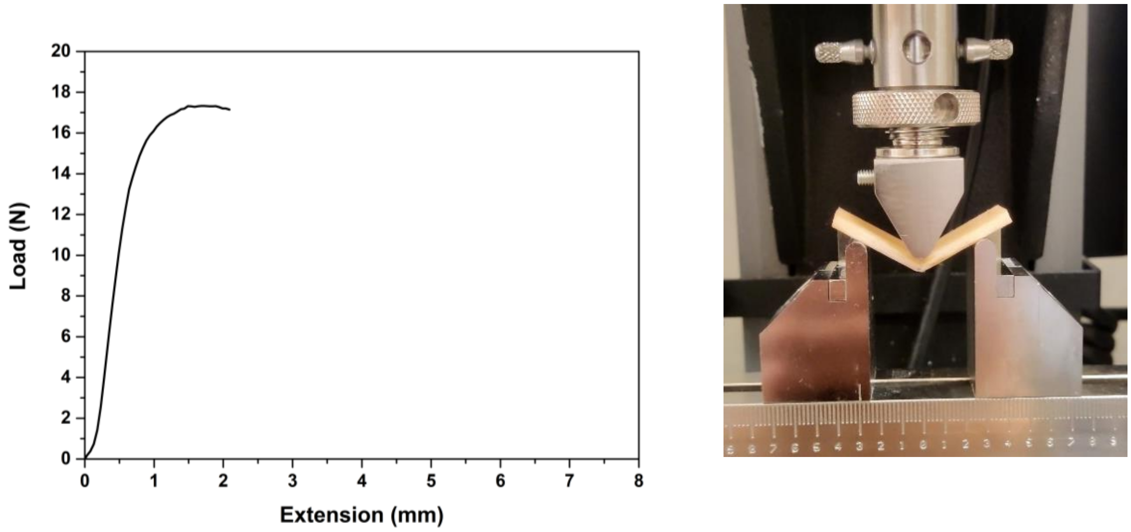

Figure S8. Left-hand side, the 3-point bending test where native wood breaks in the first cycle. Right-hand side, the broken wood piece.

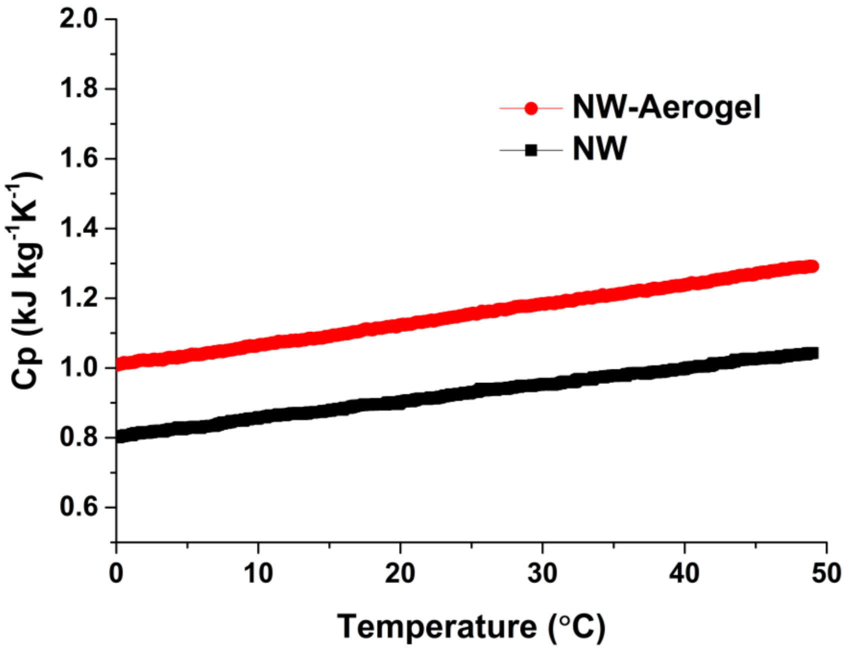

Figure S9. Specific heat capacity (Cp) of NW and NW-Aerogel

Table S2. Comparison of thermal properties and material yield for wood-based insulators in literature.

**Type & measurement conditions** (*Relative humidity %, temperature °C*)	**Material yield (%)**	**Radial λ_{radial} (W/mK)**	**Axial λ_{axial} (W/mK)**	**Reference**
Wood aerogel based on lignin and hemicellulose removal (*- %, -°C*)	35	0.028	0.12	1
Nanowood based on lignin and hemicellulose removal (*20 %, 25°C*)	~40	0.032	0.056	2
Nanowood based on lignin and hemicellulose removal (*80 %, 25°C*)	~40	0.05	0.08	2
Wood aerogel based on lignin and hemicellulose removal (*- %, -°C*)	-	0.033	-	3
Wood aerogel based on lignin removal and partial hemicellulose removal (*- %, -°C*)	-	~0.045	0.068	4
Wood aerogel based on lignin removal and partial hemicellulose removal (*- %, -°C*)	~35	0.0418	0.0613	5
Delignified wood aerogel (*50 %, 23°C*)	46	0.037	0.057	6
**NW-Aerogel (this study)** (*50 %, 23°C*)	**77**	**0.042**	**0.16**	**This study**

2. DIC Details:

DIC technique is used to measure the strain distribution on native wood and aerogel samples. Specifically, mirror-assisted multi-view digital image correlation (MV-DIC)[7] (see figure S10) that can retrieve the panoramic strain distribution is adopted to measure the highly inhomogeneous and complicated deformation behavior. Similar to the other compressive tests, DIC measurements were performed also during a radial compression in a conditioned room of 23 °C and 50% relative humidity. Cubic samples with nominal dimension of 10 x 5 x 5 mm^3 (longitudinal × radial × tangential) were compressed with preset constant strain rate of 10%/min by an Instron E1000 compression machine equipped with a 10 kN load cell. Prior to the experiments, the sample surfaces were pretreated by slight polish, followed by the decoration of thin white base coat and black random speckles. These samples were compressed by cylindrical steel heads with diameter of 10 mm. Image series were collected by a stereo-DIC system during the loading

progress to record the surface evolvement resulted from compression. To be specific, a Blue-X-Focus blue light source were fixed in front of the sample to provide uniform and constant light on the sample surface. Two Basler acA4096-30um cameras with resolution of 2168×4096 were mounted on a tripod. The two cameras with included angle of around 16.96° were distributed in a vertical plane instead of horizontally like usual. Lenses with focal length of 135 mm were fixed on the cameras by additionally inserting an extension tube. The field of view of the imaging system can well fit the sample dimension by using this configuration. Polarizers were mounted in front of the lenses and the light source. Rotating the polarizers can alleviate the specular reflection, leading to better image quality for latter analysis. Two optic quality optical mirrors with vertex angle of around 90° were fixed behind the sample. The cameras can view two surfaces simultaneously through the reflection of each mirror. Alternatively speaking, the panoramic surface can be fully captured through the reflection of the two mirrors. Speckle patterns were also partly made on the mirror surface to facilitate the subsequent panoramic measurement. During the loading progress, synchronous image series of the sample surface were recorded by the two cameras with a frame rate of 1 frame per second.

The stereo-DIC system needs to be calibrated in advance for strain measurement. A planar calibration target with 9×9 circular feature points is used. The constant space between the node points is 3 mm. More than 25 calibration image pairs were recorded by moving the calibration target to diverse poses and positions. The intrinsic and extrinsic parameters of the stereo-DIC system can be determined by processing these calibration image pairs.

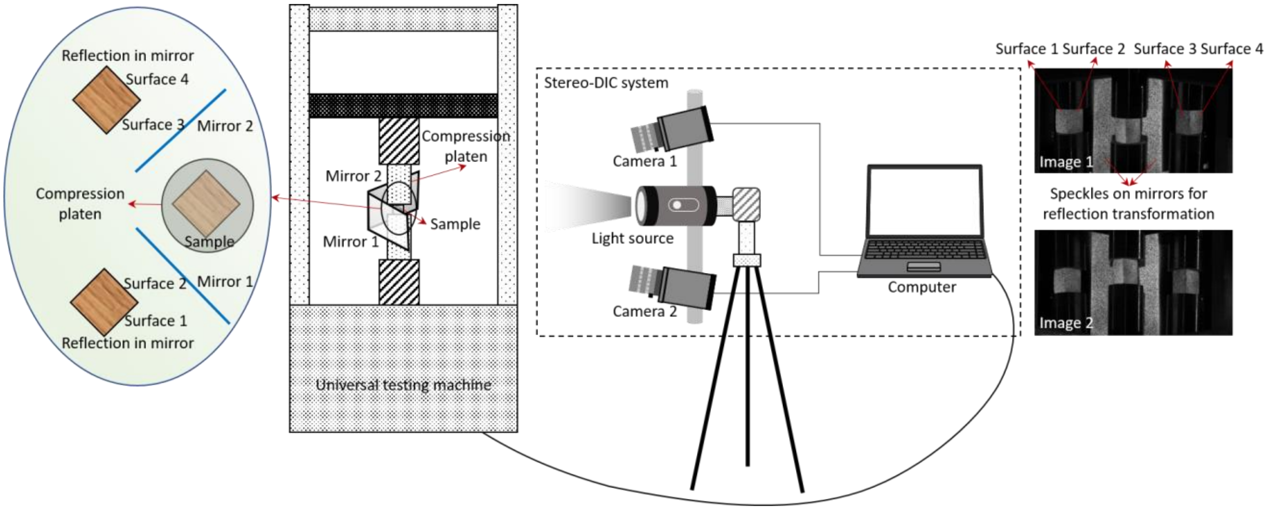

Figure S10. Illustration of mirror-assisted MV-DIC setup and measurement details

Panoramic surface deformation was retrieved by processing the recorded image pairs, see the image video 5 and video 6. Four regions of interest (Fig. S10, surface 1-4), including the two sample surfaces reflected by each mirror and the speckle patterns on the two mirrors, were specified for DIC matching. The subset size and step size between calculation points were designated to be 31 × 31 and 5 pixels, respectively. The strain was estimated from a small window size of 7 ×7 points. Aided by the reconstructed 3D shape of the two mirrors, the measurement results of the two sample surfaces, which were reflected by the two mirrors, were reflected to their real locations in front of the mirrors, leading to the panoramic strain distribution.

References

(1) Song, J.; Chen, C.; Yang, Z.; Kuang, Y.; Li, T.; Li, Y.; Huang, H.; Kierzewski, I.; Liu, B.; He, S.; Gao, T.; Yuruker, S. U.; Gong, A.; Yang, B.; Hu, L. Highly Compressible, Anisotropic Aerogel with Aligned Cellulose Nanofibers. *ACS Nano* **2018**, *12*, 140-147, DOI: 10.1021/acsnano.7b04246.

(2) Li, T.; Song, J.; Zhao, X.; Yang, Z.; Pastel, G.; Xu, S.; Jia, C.; Dai, J.; Chen, C.; Gong, A.; Jiang, F.; Yao, Y.; Fan, T.; Yang, B.; Wågberg, L.; Yang, R.; Hu, L. Anisotropic, Lightweight, Strong, and Super Thermally Insulating Nanowood with Naturally Aligned Nanocellulose. *Sci. Adv.* **2018**, *4*, DOI: 10.1126/sciadv.aar3724.

(3) Sun, H.; Bi, H.; Lin, X.; Cai, L.; Xu, M. Lightweight, Anisotropic, Compressible, and Thermally-Insulating Wood Aerogels with Aligned Cellulose Fibers. *Polymers* **2020**, *12*, DOI: 10.3390/polym12010165.

(4) Yan, M.; Fu, Y.; Pan, Y.; Cheng, X.; Gong, L.; Zhou, Y.; Ahmed, H.; Zhang, H. Highly Elastic and Fatigue Resistant Wood/Silica Composite Aerogel Operated at Extremely Low Temperature. *Compos. B. Eng.* **2022**, *230*, 109496, DOI: 10.1016/j.compositesb.2021.109496.

(5) Zhang, Q.; Li, L.; Jiang, B.; Zhang, H.; He, N.; Yang, S.; Tang, D.; Song, Y. Flexible and Mildew-Resistant Wood-Derived Aerogel for Stable and Efficient Solar Desalination. *ACS Appl. Mater. Interfaces* **2020**, *12*, 28179-28187, DOI: 10.1021/acsami.0c05806.

(6) Garemark, J.; Perea-Buceta, J. E.; Rico Del Cerro, D.; Hall, S.; Berke, B.; Kilpelainen, I.; Berglund, L. A.; Li, Y. Nanostructurally Controllable Strong Wood Aerogel toward Efficient Thermal Insulation. *ACS Appl. Mater. Interfaces* **2022**, *14*, 24697-24707, DOI: 10.1021/acsami.2c04584.

(7) Chen, B.; Pan, B. Mirror-Assisted Multi-View Digital Image Correlation: Principles, Applications and Implementations. *Opt. Lasers Eng.* **2022**, *149*, 106786, DOI: 10.1016/j.optlaseng.2021.106786.
